# Twist-Dependent In-Plane Symmetry Breaking in the WSe_2_/Black Phosphorus Interface

**DOI:** 10.1021/acs.nanolett.6c05049

**Published:** 2026-10-28

**Authors:** Yu Dong, Mao Yoshii, Ziqian Wang, Fehmi S. Yasin, Sota Kitamura, Feng Qin, Shingo Toyoda, Takahiro Morimoto, Xiuzhen Yu, Naoki Ogawa, Yoshihiro Iwasa, Toshiya Ideue

**Affiliations:** † Institute for Solid State Physics, 13143 The University of Tokyo, Kashiwa 277-8581, Japan; ‡ 13593 RIKEN Center for Emergent Matter Science (CEMS), Wako 351-0198, Japan; § Department of Applied Physics, The University of Tokyo, Tokyo 113-8656, Japan; ∥ Institutes of Innovation for Future Society, 12965 Nagoya University, Nagoya 464-8601, Japan; ⊥ Center for Nanophase Materials Sciences, Oak Ridge National Laboratory, Oak Ridge, Tennessee 37830, United States; # Tsung-Dao Lee Institute, Shanghai JiaoTong University, Shanghai 201210, China

**Keywords:** twistronics, WSe_2_/black phosphorus heterointerface, second harmonic generation, in-plane symmetry breaking, polarization

## Abstract

The control of optical responses and electronic states via the twist angle, “twistronics”, has revolutionized the research field of van der Waals heterostructures. Despite extensive advances in twisted interfaces composed of materials sharing identical or similar crystal symmetries, twist-dependent phenomena at symmetry-mismatched interfaces hosting unconventional moiré superlattices and symmetry breaking remain poorly understood. Here, we report twist-dependent in-plane symmetry breaking at a symmetry-mismatched heterointerface between WSe_2_ and black phosphorus. Employing polarization-resolved second-harmonic generation (SHG) microscopy, we characterize the systematic evolution of in-plane symmetry breaking in WSe_2_/black phosphorus interfaces by varying the twist angle. We propose the twist-dependent in-plane polarization as a possible interpretation, supported by theoretical model calculations and their good agreement with the observed evolution of the SHG response. Our findings extend twistronics to symmetry-mismatched interfaces, offering a strategy to engineer nonlinear optical responses and interfacial polarization in low-symmetry van der Waals systems.

## Graphical abstract

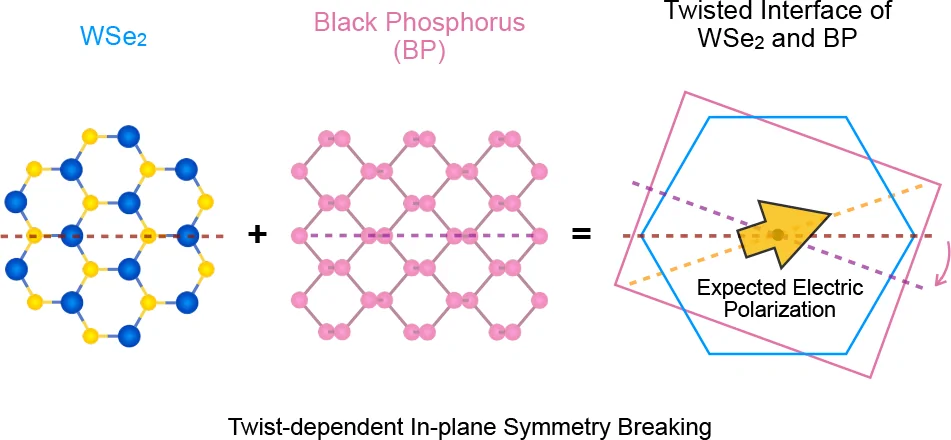

van der Waals (vdW) heterointerfaces can be fabricated with various combinations of material building blocks, stacking orders,^1–14^ and stacking angles,^15–22^ serving as a versatile platform for investigating novel physical phenomena. On one hand, symmetry engineering of the van der Waals heterointerface^6–14^ has recently been recognized as a strategic approach for realizing the functional two-dimensional systems; suitable selection of building blocks with specific symmetries can intentionally reduce the interfacial symmetry, leading to the targeted functionalities. Notably, symmetry engineering has enabled the emergence of the spontaneous photovoltaic effect and photogalvanic effect in WSe_2_/black phosphorus^6^ and WSe_2_/SiP^7^ heterointerfaces. On the other hand, the growing field of twistronics using van der Waals interfaces, initiated by the discovery of the correlated insulating state and superconductivity in “magic angle” twisted bilayer graphene,^15^ has uncovered numerous novel physical phenomena and functionalities.^16–21^ Significant progress has been made in diverse twisted moiré systems, including twisted multilayer graphene,^16^ twisted transition metal dichalcogenides (TMDs),^17–19^ and twisted two-dimensional (2D) magnets.^20,21^ However, existing twistronics research predominantly concentrates on twisted homointerfaces or heterointerfaces comprising materials with identical or similar crystal structures, for instance, the hexagonal/hexagonal lattice. Twisted vdW heterointerfaces constructed from materials possessing distinct crystal symmetries have remained largely unexplored. In this study, we systematically investigated the twisting effect in heterointerfaces constructed from WSe_2_ and black phosphorus (BP), a material combination^6^ that can host the in-plane electric polarization due to the symmetry mismatch at the interface. We fabricated twisted heterointerfaces of monolayer WSe_2_ and BP flake (typically 30–50 nm thick) using the standard dry transfer method and characterized these twisted interfaces by using the optical second-harmonic generation (SHG) microscopy, revealing a systematic evolution of the in-plane symmetry breaking. We consider the emergent in-plane electric polarization at the heterointerface and its twist-dependent evolution as a possible explanation for the observed nonlinear optical response (Figure 1a).

**Figure 1:**
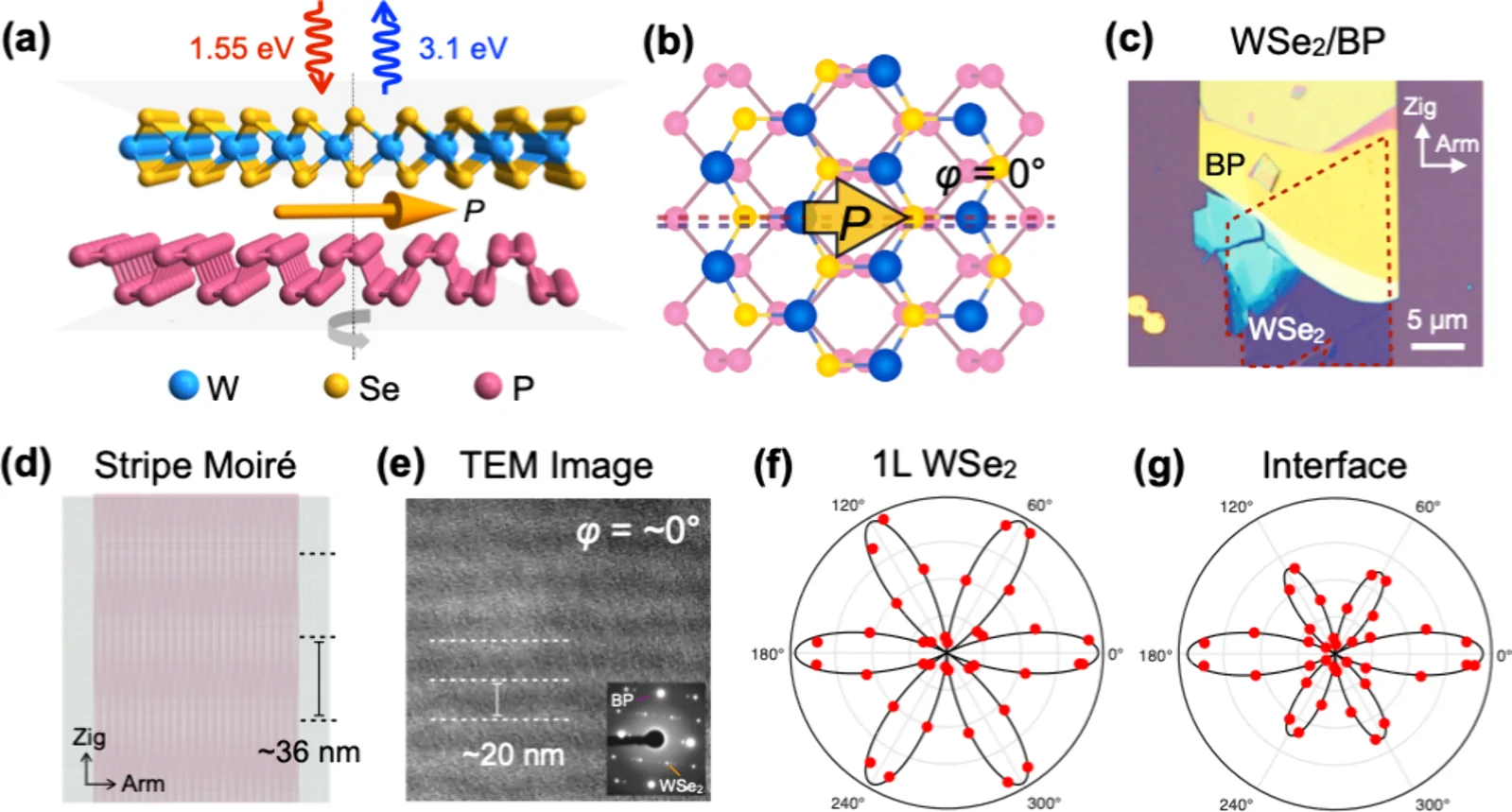
In-plane symmetry breaking and second-harmonic generation response in the WSe_2_/BP interface. (a) Schematic illustration of the emergent symmetry breaking, including in-plane electric polarization in the WSe_2_/BP interface and SHG characterization; 1.55 eV laser pulses are used to excite a 3.1 eV SHG signal. (b) Top-view schematic of the WSe_2_/BP heterointerface with the armchair directions of WSe_2_ and BP aligned in parallel (twist angle φ = 0°). (c) Optical microscope image of the 0° aligned WSe_2_/BP interface for the SHG measurement. Monolayer WSe_2_ is outlined by the red dashed line. (d) Moiré pattern of the 0° aligned WSe_2_/BP interface. The period of the stripe pattern is ∼36 nm. (e) Transmission electron microscopy image of the ∼0° aligned WSe_2_/BP interface with a stripe period of ∼20 nm, which indicates a misalignment of ∼0.7°. The inset shows the diffraction pattern. (f) Polarization-resolved SHG pattern of monolayer WSe_2_. (g) Polarization-resolved SHG pattern of the 0° WSe_2_/BP interface. The black curve represents the fitting of the SHG data. All SHG patterns are normalized and plotted on the same intensity scale for direct comparison.

As a typical transition metal dichalcogenide, WSe_2_ has a crystal structure with 3-fold rotational symmetry and three mirror planes along armchair directions. In contrast, BP possesses 2-fold rotational symmetry and a single mirror plane along the armchair direction. The symmetry mismatch between the 3-fold symmetry of WSe_2_ and the 2-fold symmetry of BP breaks the overall rotational symmetry at the WSe_2_/BP heterointerface. When the armchair direction of WSe_2_ and BP are aligned (defined as twist angle φ = 0°), the mirror symmetry along the common armchair direction is preserved, giving rise to an in-plane electric polarization along this mirror plane,^6^ as illustrated in Figure 1b. Different from conventional ferroelectric materials, the orientation of the emergent polarization at WSe_2_/BP interface is determined by the triangular lattice orientation of WSe_2_, which is not switchable once the heterointerface stacked (see Figure S1). The bulk photovoltaic effect that potentially reflects this emergent electric polarization has been recently observed specifically at interfaces between materials with 3- and 2-fold rotational symmetries.^6,10–14^ The bulk photovoltaic effect was also observed in a 15° twisted WSe_2_/BP interface (Figure S2), providing complementary evidence for the existence of interfacial polarization. Due to the lattice mismatch between WSe_2_ and BP, a unique stripe-like moiré superlattice^22^ (with an estimated period of ∼36 nm for the well-aligned interface), distinct from conventional hexagonal moiré patterns, emerges at the WSe_2_/BP interface (see Figure 1d). The periodic stripe pattern is oriented parallel to the mirror plane and may influence the interfacial anisotropy and modulate electric polarization. For instance, the interfacial polarization may exhibit a periodically modulated intensity, and possibly its orientation, as well, following the stripe moiré superlattice (Figure S3). The expected stripe-like moiré pattern is observed by high-resolution transmission electron microscopy (TEM) in a well-aligned WSe_2_/BP interface sample prepared on a SiN membrane (Figure 1e), with a period of ∼20 nm. We note that the small discrepancy of the moiré period may be due to the inevitable misalignment (estimated to be ∼0.7°). More details about period estimation of the moiré superlattice in the WSe_2_/BP interface can be found in Figures S4 and S5. Since SHG measures the spatially averaged nonlinear optical response over the selected region of interest, the local moiré superlattice may also affect the spatially averaged SHG signal.

We first studied the well-aligned (φ = 0°) WSe_2_/BP interface and examined the possible effect of the emergent in-plane electric polarization on the SHG response. An optical image of a well-aligned WSe_2_/BP device is shown in Figure 1c. Second-harmonic generation microscopy was employed to investigate in-plane symmetry breaking of this interface. In general, SHG is known to be a sensitive probe of symmetry breaking in solids,^23–26^ and the stacking-dependent SHG has been studied in graphene^27^ and homointerfaces.^28–30^ In the SHG measurements, linearly polarized laser pulses (1.55 eV, 1.5 μJ) were irradiated normally on the entire sample through an objective lens at room temperature (see Figure S6), such that only the in-plane nonlinear susceptibility tensor components and the corresponding polarization effects were detected. No sample damage occurs under this laser excitation power. SHG signals were recorded under a parallel configuration, where the linear light polarization of excitation and detection are set to be parallel. The polarization angle-dependent SHG intensities averaged from the region of interest in monolayer WSe_2_ and the WSe_2_/BP heterointerface are depicted in panels f and g, respectively, of Figure 1. Note that BP is SHG silent due to its centrosymmetric crystal structure. While monolayer WSe_2_ shows a homogeneous 6-fold SHG pattern, fitted by the formula *I* ∝ *A*_WSe_2__|cos­(3θ)|^2^, reflecting its intrinsic 3-fold rotational symmetry, the WSe_2_/BP heterointerface exhibits a distinct 2-fold SHG pattern with the peaks enhanced along the mirror plane (expected electric polarization) direction. This observation indicates the in-plane symmetry breaking at the WSe_2_/BP heterointerface and is consistent with a contribution from the interfacial electric polarization. The observed experimental pattern is fitted well by the phenomenological formula *I* ∝ *A*|cos­(3θ) + *P*_0_ cos­(θ – θ_0_)|^2^, as indicated by the black line in Figure 1g, where *P*_0_ and θ_0_ are the parameters characterizing the magnitude and direction, respectively, of the in-plane symmetry breaking. For a well-aligned (φ = 0°) WSe_2_/BP interface, orientation parameter θ_0_ is aligned nearly along the expected polar direction (0°), being consistent with our expectation. The reproducibility of the SHG response from the WSe_2_/BP heterointerface can be found in Figures S7 and S8.

The observed anisotropic polarization dependence of the SHG signal can be attributed to the intrinsic electric polarization, although the extrinsic interference effects^31,32^ also play a role in the present sample of the WSe_2_/BP heterostructure prepared on SiO_2_ (300 nm)/Si substrates. As the excitation laser pulses propagate through multiple layers, including the WSe_2_ monolayer, the BP flake, and the underlaying SiO_2_ layer, and are reflected at each interface, the interference effects modulate the actual excitation power at the heterointerface. Meanwhile, the SHG signals generated at the WSe_2_/BP interface also go through such multiple interference effect, as illustrated in Figure 2a. Considering that BP is a birefringent material, in which the refractive index differs along zigzag and armchair directions, the interference effect could cause an anisotropic SHG response. By using the reported refractive indices of BP^33,34^ and determining the nonlinear Maxwell equations^35,36^ within the multilayer structure, we simulate the reflected SHG response of the heterointerface. We assume the BP thickness to be ∼40 nm, consistent with that of the sample shown in Figure 1c. The SHG simulation of monolayer WSe_2_ (exhibiting a homogeneous 6-fold SHG pattern) incorporating only the interference effect is obtained in Figure 2b, which shows an anisotropic pattern with peak suppression at 0° and 180° (the expected polar direction). This result fundamentally contrasts with the experimental SHG data shown in Figure 1g, where an enhancement is observed along the polar direction. This discrepancy indicates that interference alone cannot account for the experimental results. By including the intrinsic electric polarization in addition to the interference effects, the experimentally observed SHG pattern can be reproduced, as shown by the simulation in Figure 2c. Although we consider polarization to be a reasonable interpretation of this additional contribution, we remain open to other possible mechanisms that may also contribute. Further SHG simulations on BP thickness and interface twist angle dependence are presented in Figures S9–S11. Note that, although the BP thickness can in principle influence the interference effect, the range of BP thicknesses investigated in this work yields a qualitatively consistent contribution from the birefringent interference effect. As a result, the conclusions presented here remain robust against moderate variations in BP thickness. These results support the important role of intrinsic in-plane symmetry breaking, including the possible contribution of electric polarization, in the SHG response at the WSe_2_/BP interface.

**Figure 2:**
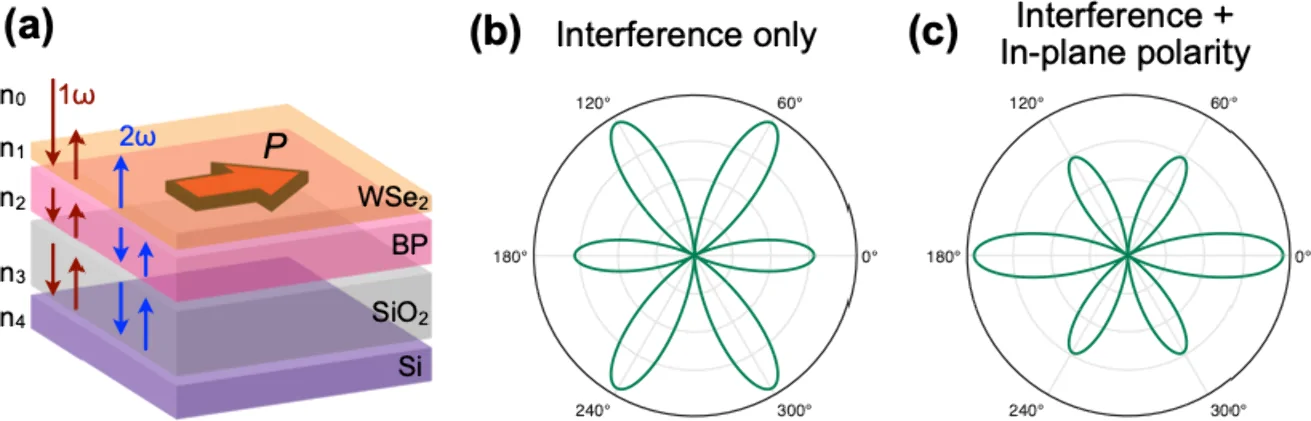
Effects of multiple interference and interface electric polarization on the SHG pattern in the WSe_2_/BP heterointerface. (a) Schematics of the multiple interference of fundamental and second-harmonic waves during the SHG process in the WSe_2_/BP heterostructure on a SiO_2_/Si substrate. The red and blue arrows represent the fundamental and second-harmonic waves, respectively. The in-plane electric polarization is indicated by the large orange arrow. (b) Simulated SHG pattern considering only the interference effect. (c) Simulated SHG pattern incorporating both the interference effect and the electric polarization effect.

We further investigated the twisted WSe_2_/BP interface with various twist angles (φ = 5–30°, in 5° increments (see sample images in Figure S12)). Introducing a twist angle between WSe_2_ and BP breaks the mirror symmetry along the initially aligned armchair direction. The SHG patterns for each twisted heterointerface were measured and are summarized in the top row of Figure 3. These results reveal that the SHG intensity peaks along 0° and 180° remain consistently enhanced. In the meantime, the peak intensities at 120° and 300° gradually increase with the twist angle of the interface, leading to a systematic change from the SHG pattern observed at the 0° interface. It is noted that all of the anisotropic SHG patterns for twist angles φ of 5–25° are consistent with the broken mirror symmetry at the twisted WSe_2_/BP interface. The evolution of moiré patterns in the twisted interface can be found in Figure S5, and the corresponding TEM results can be found in Figures S13–S15.

**Figure 3:**
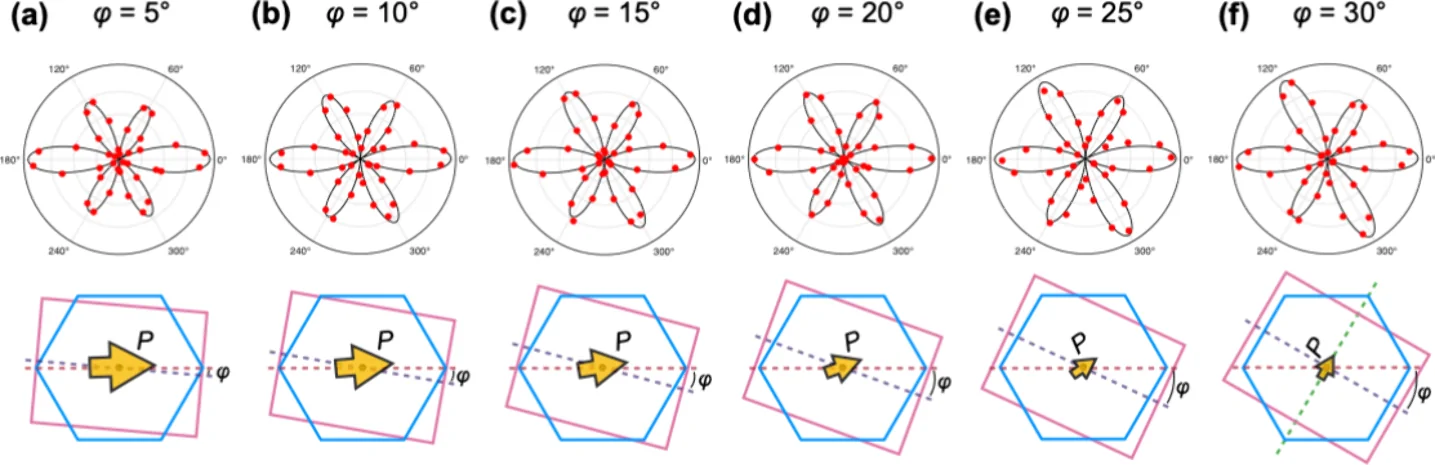
Polarization-resolved SHG patterns in twisted WSe_2_/BP interfaces. (a–f) Evolution of the SHG response in WSe_2_/BP heterointerfaces with varying interface twist angles (φ = 0–30° in 5° increments). For each twist angle, the corresponding experimentally observed SHG pattern (top row) and schematics of calculated interfacial electric polarization (bottom row) are presented. The red and purple dashed lines highlight the armchair directions of WSe_2_ and BP, respectively. The green dashed line indicates the glide reflection symmetry direction. The orange arrows indicate the direction of the calculated in-plane electric polarization in the twisted interface. The size of the arrows corresponds to the magnitude of electric polarization. All SHG data are normalized and plotted on a unified intensity scale for direct comparison.

Interestingly, when φ = 30°, where the armchair direction of WSe_2_ aligns parallel with the zigzag direction of BP, the polarization-resolved SHG intensity exhibits a distinct 2-fold symmetric pattern. Unlike the mirror symmetric patterns along 0° axis in the case of a well-aligned interface (φ = 0°), the SHG pattern of the 30° twisted interface is mirror symmetric along the 60° and 150° axes. The SHG result of the 30°-twisted WSe_2_/BP interface in the cross configuration, where the linear light polarization of excitation and detection are set to be perpendicular, also shows a mirror symmetric pattern along the 60° axis (Figure S16). This result can be understood by the emergence of glide reflection (quasi-mirror) symmetry at the 30° twisted WSe_2_/BP interface, along the coaligned armchair (WSe_2_) and zigzag (BP) directions (green dashed line in Figure 3). Meanwhile, the in-plane electric polarization of the 30° twisted WSe_2_/BP interface is expected to align along the glide symmetry direction. The two high-symmetry configurations, achieved at 0° and 30° twisted WSe_2_/BP interfaces, define the in-plane polarization orientation. Therefore, the evolution of the SHG patterns with twist angle can be intuitively interpreted as a continuous rotation of the polarization direction along a nonlinear trajectory between these two limiting configurations. At intermediate twist angles, the interface no longer preserves an exact mirror or glide symmetry, resulting in gradual distortion of the SHG anisotropy pattern between the 0° and 30° twist angle limits. Note that the existence of in-plane polarization in the twisted WSe_2_/BP heterointerface is supported by the BPVE results observed in a 15° twisted heterointerface (Figure S2). To further elucidate the twist angle-dependent SHG response in the WSe_2_/BP heterointerface, we developed an effective tight-binding model^6,37^ of the twisted interface to calculate the in-plane electric polarization and second-order susceptibility tensor (more details about the model in the Supporting Information and the calculated SHG polarization patterns in Figure S17). The in-plane electric polarization evolution calculated from our model as a function of the interface twist angle is shown in the schematics at the bottom row of Figure 3. The magnitude and orientation of the in-plane electric polarization change with an increase in the interface twist angle (see Figure 4a, as well).

**Figure 4:**
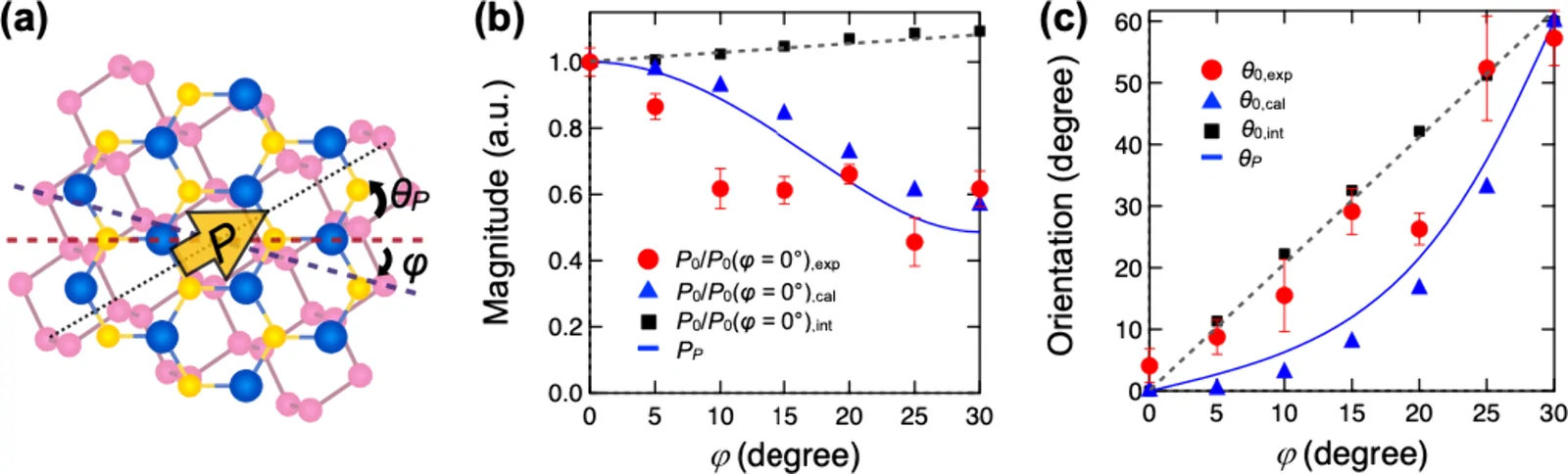
Proposed twist-dependent in-plane electric polarization in WSe_2_/BP interfaces. (a) Schematics of the expected in-plane electric polarization evolution in twisted WSe_2_/BP heterointerfaces. φ and *θ*_P_ indicate the twist angle and the direction of the electric polarization, respectively. (b and c) Experimentally/theoretically estimated parameters describing in-plane symmetry breaking (magnitude parameters (b) and orientation parameters (c), respectively) and their comparison with the calculated in-plane electric polarization as a function of twist angle. The black, red, and blue points represent the values estimated from the fittings of the SHG simulation considering only the interference effect, experimentally observed SHG patterns, and calculated SHG patterns, respectively. The red error bar indicates the standard error estimated from the fitting. The blue lines indicate the electric polarization directly calculated by using the tight-binding model. The gray dashed line is a guide for the eyes to highlight the evolution trend of interference-simulated SHG patterns as a function of twist angle.

To quantitatively discuss the evolution of the in-plane symmetry breaking at the interface and compare it with the calculated interfacial electric polarization, we employed the aforementioned phenomenological fitting formula: *I* ∝ *A*|cos­(3θ) + *P*_0_ cos­(θ – θ_0_)|^2^. Parameters *P*_0_ and θ_0_ were extracted from experimentally observed (*P*_0,exp_) and theoretically (*P*_0,cal_) calculated SHG patterns in twisted WSe_2_/BP interfaces (red and blue data points in panels b and c of Figure 4). In-plane electric polarization, directly calculated from the polarization current (blue line), is simultaneously displayed. For comparison, *P*_0_ and θ_0_ derived from the simulated SHG patterns considering only interference effects (*P*_0,int_) were also plotted (black data points). The distinct competition between the interfacial polarization effect and birefringent interference effect contributes differently to the evolution of the magnitude and orientation parameters extracted from the SHG fitting.

Figure 4b shows the twist angle dependence of the extracted magnitude parameters unified by their values at the 0° interface, *P*_0,int_/*P*_0,int_ (φ = 0°), *P*_0,exp_/*P*_0,exp_ (φ = 0°), and *P*_0,cal_/*P*_0,cal_ (φ = 0°), along with the calculated electric polarization magnitude *P*_P_/*P*_P_ (φ = 0°). We can notice that the trends are qualitatively different between *P*_0,int_/*P*_0,int_ (φ = 0°) and the other cases. *P*_0,int_/*P*_0,int_ (φ = 0°) shows the small increasing behavior, while the others are suppressed with the twist angle. We confirmed that the trend of *P*_0,int_/*P*_0,int_ (φ = 0°) is not affected by changing simulation parameters such as the refractive index and thickness of the black phosphorus from the current values (see Figure S11). This qualitative difference strongly indicates that observed data cannot be explained by considering only the interference effect, reinforcing the previous argument of Figure 2. The decreasing trend of the magnitude parameters extracted from the experimental data is quite consistent with our theoretical calculation considering the electric polarization effect. Both the calculated electric polarization magnitude and the magnitude parameter extracted from calculated SHG patterns decrease nonlinearly by more than 40% as the interface twist angle increases from 0° to 30°. When both interference and polarization effects are considered simultaneously, the suppression originating from the interfacial polarization dominates over the relatively weak interference contribution. As a result, experimentally extracted magnitude *P*_0,exp_ shows good agreement with the polarization-based theoretical calculations and directly calculated polarization magnitude *P*_P_.

Figure 4c shows the twist angle variation of the orientation parameters extracted from the simulated (θ_0,int_), experimental (θ_0,exp_), and theoretical (θ_0,cal_) SHG patterns, alongside the directly calculated electric polarization orientation (*θ*_P_). For the simulated SHG patterns considering only the interference effect, the extracted orientation parameter increases linearly from 0° to 60° as a function of twist angle (gray dashed line). The in-plane orientation of the calculated electric polarization also rotates from 0° to 60°, directions determined by the mirror and glide reflection symmetry, in twisted WSe_2_/BP interfaces. However, in contrast to the linear parameter change caused by the interference simulation, the electric polarization orientation gradually rotates from 0° toward 60° with the twist angle, following a nonlinear trajectory, as highlighted by blue line in Figure 4c. The orientation parameters extracted from the experimentally observed SHG patterns exhibit an intermediate trajectory between the interference simulation (black dashed line) and electric polarization calculation (blue line), with a nonlinear twist angle dependence. Compared with the magnitude parameter, the orientation parameter is therefore more sensitive to the coexistence and competition between birefringent interference and interfacial polarization effects. Note that the possible modulation of the in-plane polarization orientation by the stripe-like moiré in twisted interfaces may also contribute to the deviation between extracted parameter θ_0_ and theoretical calculated polarization orientation *θ*_P_, since SHG measures a spatially averaged signal. The experimentally observed nonlinear evolution of θ_0_ reflects the combined influence of these two mechanisms, rather than purely following either contribution individually. Based on the analyses above, we consider twist-dependent in-plane electric polarization to be a possible mechanism underlying the twist-dependent in-plane symmetry breaking observed by SHG at the WSe_2_/BP interface.

We investigated the evolution of the in-plane symmetry breaking in twisted WSe_2_/BP heterointerfaces through the optical SHG microscopy. We first examined the well-aligned (0°) WSe_2_/BP interface and found that an additional contribution beyond the optical interference effect is required to explain the observed anisotropic SHG pattern, particularly the enhanced peaks along the polar direction. We consider the interfacial in-plane electric polarization to be a possible origin of this additional contribution. The SHG response of twisted WSe_2_/BP interfaces exhibits a systematical evolution with twist angle. Our effective tight-binding calculations predict a twist-dependent evolution of the interfacial in-plane polarization. Through the fitting of the SHG patterns and comparison with the theoretical calculations, we propose that the twist-dependent rotation and magnitude modulation of the in-plane electric polarization provide a possible explanation for the observed evolution of the SHG response. These results show that the twist angle provides an effective way to modify the in-plane symmetry and nonlinear optical response at symmetry-mismatched van der Waals interfaces and may also provide a route to engineer interfacial polarization. Twist-dependent symmetry breaking including emergent polarization may be a general feature of symmetry-engineered heterointerfaces. More broadly, twist-dependent optical responses and transport anisotropy may provide new opportunities for designing reconfigurable optoelectronic and quantum devices. In addition, the unique stripe moiré superlattice and emergent in-plane polarization may offer exciting opportunities for exploring anisotropic excitonic phenomena (Figure S18) and anisotropic Hubbard physics in symmetry-mismatched van der Waals heterointerfaces.

## Supplementary Material



## Data Availability

The data that support the findings of this study are available from the corresponding author upon request.

## Notes

Experimental details, photocurrent results, moiré period estimation, schematics of the measurement setup, reproducibility results, interference effect simulation, additional TEM results, SHG responses in cross configuration, details of the model calculation, photoluminescence results, Raman spectrum characterization, and Figures S1–S19 (PDF)

## 7

Department of Physics, Kyoto University, Kyoto 606-8502, Japan
