# Supplementary material for: Twist-Dependent In-Plane Symmetry Breaking in the WSe2/Black Phosphorus Interface
Source: Nano Lett. 2026 Oct 28;26(44):15507–13. doi: 10.1021/acs.nanolett.6c05049 (PMC13761675; doi:10.1021/acs.nanolett.6c05049)
Supplement: Supplementary file 1 [file nl6c05049_si_001.pdf]

Supporting Information

Twist-Dependent In-Plane Symmetry Breaking in the WSe_2/Black Phosphorus Interface

Yu Dong, Mao Yoshii, Ziqian Wang, Fehmi S. Yasin, Sota Kitamura, Feng Qin, Shingo Toyoda, Takahiro Morimoto, Xiuzhen Yu, Naoki Ogawa, Yoshihiro Iwasa and Toshiya Ideue**

* ideue@issp.u-tokyo.ac.jp

* iwasay@riken.jp.

1. Experimental details

2. The stacking-determined polarization domain in WSe_2/BP heterointerface

3. Photocurrent response from twisted WSe_2/BP heterointerface

4. Influence of the stripe moiré superlattice on polarization and estimation of the superlattice period in twisted WSe_2/BP interfaces

5. The optical path of SHG measurement set-up

6. Reproducibility of SHG response in WSe_2/BP interface

7. Interference effect: simulation method and additional simulation results

8. Optical images of twisted WSe_2/BP samples

9. Transmission electron microscopy characterization on twisted WSe_2/BP interfaces

10. SHG response of WSe_2/BP interface in parallel and cross configurations

11. Details about tight-binding model for twisted WSe_2/BP interface and calculation method

12. Photoluminescence response of WSe_2/BP interface

13. Raman characterization of the crystal orientation in black phosphorus

14. Discussion about strain in WSe_2/BP heterointerface

1. Experimental details

Sample preparation: BP and monolayer WSe_2 flakes were mechanically exfoliated onto a SiO_2/Si substrate and a polydimethylsiloxane (PDMS) sheet, respectively. BP crystals were sourced from Smart-elements, and WSe_2 crystals were purchased from hq graphene. The number of layers in the WSe_2 flakes was determined based on optical contrast in the image obtained by optical microscope (Olympus BXFM-A). Monolayer WSe_2 was transferred onto the BP flake at a predetermined twist angle using an all-dry transfer method[S1]. All exfoliation and transfer procedures were conducted inside a glove box filled with N_2. The crystal direction of each flake was identified by examining the edge of the flake[S2], and BP crystal direction was further verified through angle-resolved Raman spectroscopy[S3], as shown in Figure S19. After transfer, the WSe_2/BP heterointerface was annealed at 220 °C for 2 hours in an N_2 atmosphere to improve interface quality.

Room-temperature SHG measurement: The samples were mounted on a sample holder for SHG measurements. A Ti:Sapphire amplifier (1 kHz, 100 fs) was used to generate fundamental laser pulses at 1.55 eV. The laser pulses were directed to the sample using a pseudo-Köhler illumination geometry through an objective lens (Olympus SLMPLN 50x). The SHG signal at 3.1 eV was collected and imaged using a charge-coupled device (CCD) camera (Princeton Instruments PIXIS: 1024B). The pulse energy was 1.5 μJ, and the exposure time for each SHG image is 2 minutes. For linear polarization-resolved SHG measurements, the polarization directions of the incident and detected light were kept parallel. The incident polarization was controlled by rotating a half-wave plate from 0° to 180° in defined steps. SHG intensities were obtained by averaging pixel values within defined circular regions of interest in the captured images. Background noise, primarily due to CCD dark counts, was subtracted to isolate the SHG signal from the sample.

Raman measurement: Raman measurements were performed in a commercial Raman spectrometer (inVia TM confocal Raman microscope, Renishaw). The sample was mounted on a rotational mount. The linear polarization dependence of Raman spectrum was measured by rotating the sample.

Transmission electron microscopy measurement: We carried out transmission electron microscopy (TEM) observations on aligned and twisted WSe_2/BP heterointerfaces by using a commercial TEM (JEM 2800, JEOL). All TEM images and the corresponding electron diffraction patterns were obtained in normal mode at an accelerating voltage of 100 kV. The samples for TEM measurements were prepared on silicon nitride (window region thickness: 50 nm) membranes designed for TEM using a procedure similar to that described in the sample preparation section.

Photocurrent-mapping measurement: Photocurrent-mapping measurements were carried out using a self-constructed piezo stage mapping system (Thorlabs NF15AP25/M). The sample was illuminated using a He-Ne laser (632.8 nm). The laser beam was focused on the sample surface at normal incidence through a ×100 Olympus objective. The linear polarization of the laser was created using a Glan–Taylor prism placed directly after the laser. The spontaneous photocurrent was measured using a source meter (Keithley 2636B) under ambient conditions.

Photoluminescence measurement: The photoluminescence (PL) measurements were also carried out in a liquid-Helium flowing type cryostat (Cryo-Industry) with a 632.8 nm laser. The PL emission was collected using a HORIBA spectrometer (iHR 320) equipped with a HORIBA Symphony II CCD camera.

SHG fitting formula: In case of $\varphi = 0°$, there is only a mirror symmetry. Based on this symmetry constraint, the second-order nonlinear susceptibility tensor components that contribute to the SHG signal under our measurement configuration are $\chi_{xxx}, \chi_{xyy}, \chi_{yxy}$. Accordingly, the polarization-resolved SHG intensity can be expressed as: $I \propto |P_x \cos\theta + P_y \sin\theta|^2 = |\frac{\chi_{xxx}-2\chi_{yxy}-\chi_{xyy}}{4}\cos 3\theta + \frac{3\chi_{xxx}+2\chi_{yxy}+\chi_{xyy}}{4}\cos\theta|^2$. This expression can be generalized and written into the phenomenological fitting formula used in the main text for all twisted WSe_2/BP heterointerfaces: $I \propto A|\cos(3\theta) + P_0\cos(\theta - \theta_0)|^2$. Note that though the symmetry of twisted interface is further reduced and additional tensor components will be allowed, the phenomenological fitting formula remains the same; their effect is incorporated in the fitting parameters P_0 and θ_0. From the good consistency between the fitting of experimental data and the fitting of theoretical calculation results, we confirmed the validity of using the phenomenological formula for twisted interfaces SHG pattern fitting.

2. The stacking-determined polarization domain in WSe_2/BP heterointerface

The expected emergent in-plane polarization in WSe_2/BP heterointerface differs fundamentally from that in conventional ferroelectric materials. In the present system, the in-plane polarization originates from the rotational-symmetry mismatch between the trigonal lattice of WSe_2 and the rectangular lattice of BP. Once the heterointerface is formed, the polarization orientation is determined by the lattice configuration and therefore does not exhibit switchability. As a result, the WSe_2/BP heterointerface is expected to behave as a single polarization domain, whose direction is fixed by the interface geometry, as shown in Figure S1.

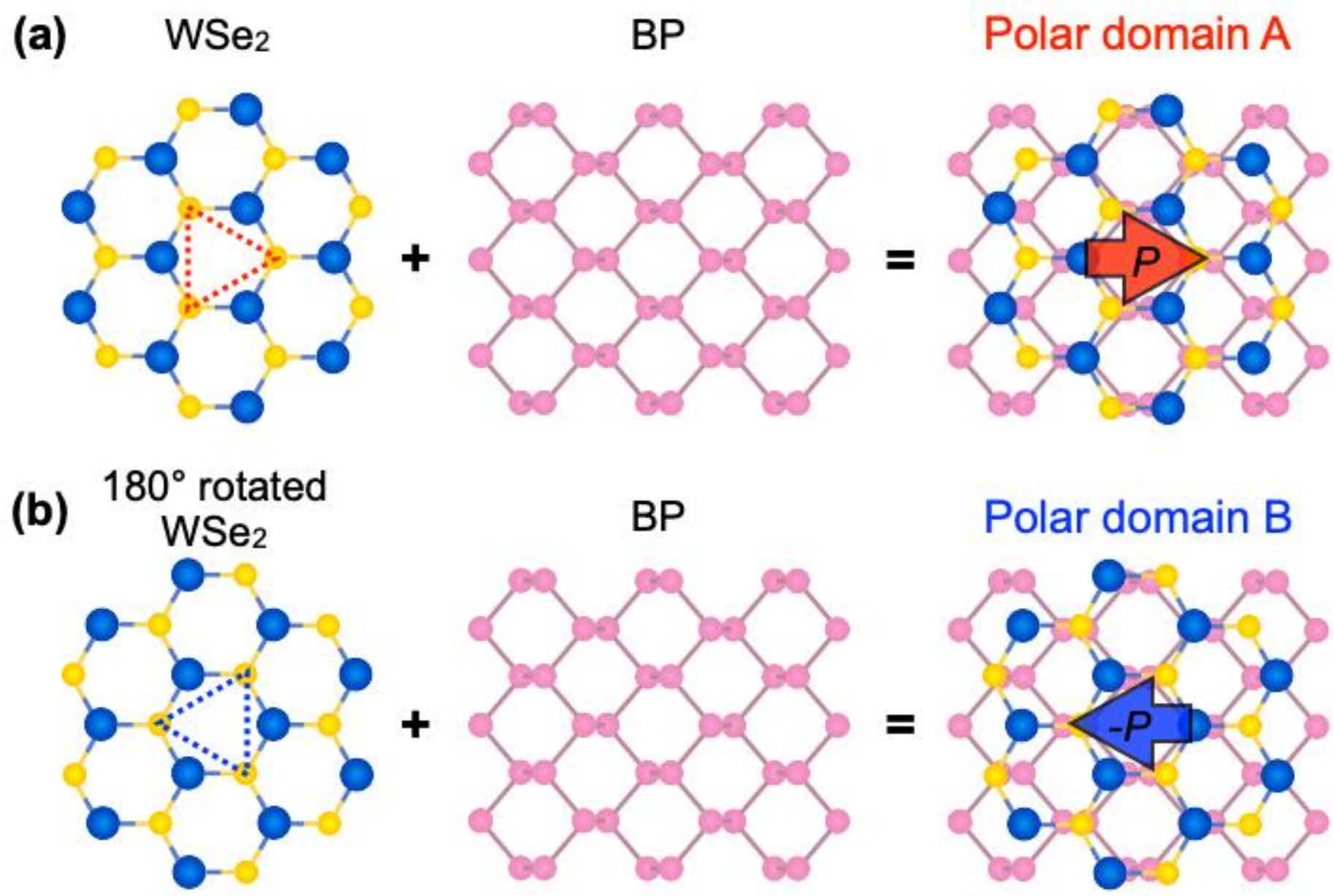

Figure S1. Expected stacking-determined polarization domains in WSe_2/BP interface. (a), Schematic illustration of WSe_2, BP lattices and polarization domain A of WSe_2/BP heterointerface. (b), Schematic illustration of WSe_2 after a 180° rotation, BP lattices and polarization domain B in WSe_2/BP heterointerface. The direction of in-plane polarization is determined by the stacking configuration of WSe_2 trigonal lattice relative to BP lattice.

3. Photocurrent response from twisted WSe_2/BP heterointerface

We performed BPVE measurements on a 15° twisted WSe_2/BP heterointerface, as shown in Figure S2. The armchair directions of WSe_2 and BP are indicated by the red and purple dashed lines in Figure S2a, while the expected in-plane polarization direction is highlighted by the orange dashed line. Among different electrode directions (E6-E10, E5-E9 and E2-E8), the strongest photovoltaic response is observed along direction E6-E10 (Figure S2b), which is closest to the expected polarization direction. The responses along E5-E9 (Figure S2c) and E2-E8 (Figure S2d) are significantly weaker, consistent with the directional nature of the interfacial polarization. These BPVE measurements provide complementary support for the existence of in-plane polarization in the 15° twisted WSe_2/BP heterointerface, although the precise polarization orientation cannot be resolved from the present electrode geometry.

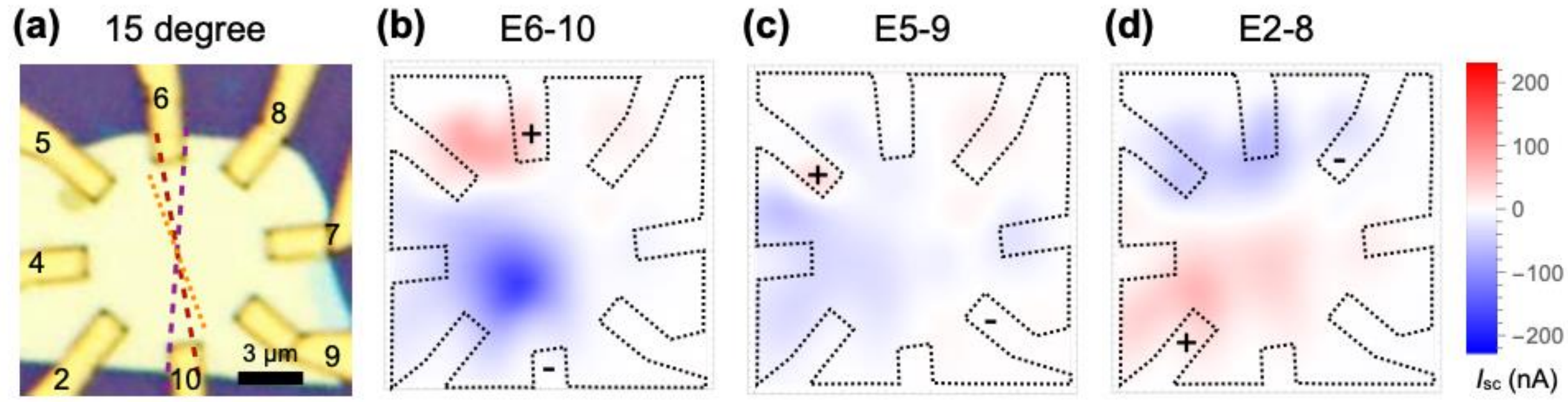

Figure S2. Photocurrent mapping from a 15° twisted WSe_2/BP interface. (a) Optical microscope image of the 15° twisted WSe_2/BP device. The red and purple dashed lines indicate the armchair direction of WSe_2 and BP, respectively, while the orange dashed line marks the expected in-plane polarization direction. (b-d) Photocurrent mapping measured along the E6-E10 (b), E5-E9 (c), and E2-E8 (d) electrode directions.

4. Influence of the stripe moiré superlattice on polarization and estimation of the superlattice period in twisted WSe_2/BP interfaces

The moiré pattern in the symmetry-mismatched WSe_2/BP interface possesses a highly anisotropic stripe-like geometry originating from the rotational-symmetry mismatch between the trigonal lattice of WSe_2 and the rectangular lattice of BP. In the small-twist-angle regime, the stripe pattern is oriented approximately parallel to the mirror symmetry direction, while the bright and dark stripe regions are periodically modulated along the perpendicular direction. The emergent in-plane polarization at the WSe_2/BP interface originates from the symmetry mismatch and interfacial coupling between two materials. Since the stripe moiré superlattice spatially modulates the local atomic registry and interlayer coupling strength, it is expected to induce a corresponding spatial modulation of the interfacial polarization. In this picture (see Figure S3), different regions within the stripe-like moiré may exhibit locally varying polarization magnitudes and possibly orientation (especially in the twisted heterointerface), leading to an inhomogeneous polarization landscape across the interface. As mentioned in the main text, since SHG measures the spatially averaged nonlinear optical response over the selected region of interest, the local moiré superlattice may also affect the spatially averaged SHG signal.

We estimated the period of moiré superlattice in WSe_2/BP interface by considering the lattice mismatch between two materials. The unit cell of WSe_2 is redefined for the convenience of superlattice period estimation, as shown in Figure S4a. The vectors along zigzag and armchair directions are a_1 = 0.328 nm and a_2 = 0.568 nm. The unit cell of BP is shown in Figure S4b, with two vectors of b_1 = 0.331 nm and b_2 = 0.437 nm. The reciprocal lattice vectors of WSe_2 and BP are defined as $\boldsymbol{g}_1 = \frac{2\pi}{a_1}(1,0)$, $\boldsymbol{g}_2 = \frac{2\pi}{a_2}(1,0)$ and $\boldsymbol{p}_1 = \frac{2\pi}{b_1}(\cos\varphi, \sin\varphi)$, $\boldsymbol{p}_1 =$

$\frac{2\pi}{b_1}(-\sin\varphi, \cos\varphi)$. Here, φ is the twist angle of the interface. The moiré reciprocal lattice vectors are calculated by the difference of WSe_2 and BP reciprocal lattice vectors as $\boldsymbol{k}_i = \boldsymbol{g}_i - \boldsymbol{p}_i$ ($i = 1, 2$). Thus, the moiré periods are calculated by $\lambda_i = \frac{2\pi}{|\boldsymbol{k}_i|}$, more specifically $\lambda_1 = \frac{b_1}{\sqrt{\frac{b_1^2}{a_1^2} - \frac{2b_1\cos\theta}{a_1} + 1}}$ and $\lambda_2 = \frac{b_2}{\sqrt{\frac{b_2^2}{a_2^2} - \frac{2b_2\cos\theta}{a_2} + 1}}$. From this method, the moiré periods of well aligned (0°) WSe_2/BP interface are estimated to be $\lambda_1^{0^\circ} = 36.2$ nm and $\lambda_2^{0^\circ} = 1.89$ nm, as indicated by the dashed lines in Figure S4c. The moiré periods decrease rapidly with the increase of twist angle. For example, 3° twisted interface has periods $\lambda_1^{3^\circ} = 6.2$ nm, $\lambda_2^{3^\circ} = 1.85$ nm, and 10° twisted interface has periods $\lambda_1^{10^\circ} = 3.13$ nm, $\lambda_2^{10^\circ} = 1.76$ nm. The observed period of stripe-like moiré pattern (~20 nm) in the well-aligned WSe_2/BP is close to the above estimation. Deviation from the ideal estimated value might result from the unintentional twist angle error introduced in the fabrication process. The varying stripe-like moiré superlattices as a function of the interface twist angle are shown in Figure S5. As the twist angle increases, the stripe orientation in the moiré pattern tilts away from the horizontal armchair direction, and the stripe unit cell size rapidly decreases.

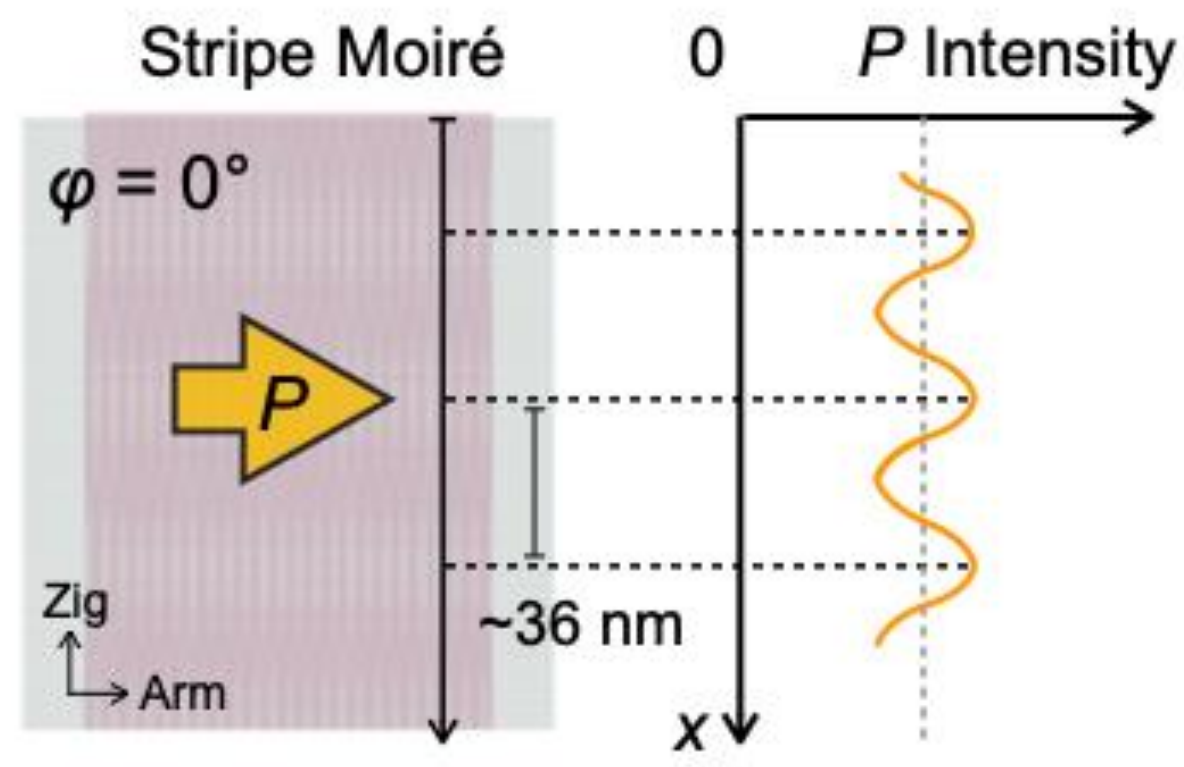

Figure S3. Schematic illustration of the stripe moiré superlattice at WSe_2/BP heterointerface (left), and the expected spatial distribution of polarization intensity along the zigzag direction (right), exhibiting the same periodicity as the stripe moiré pattern.

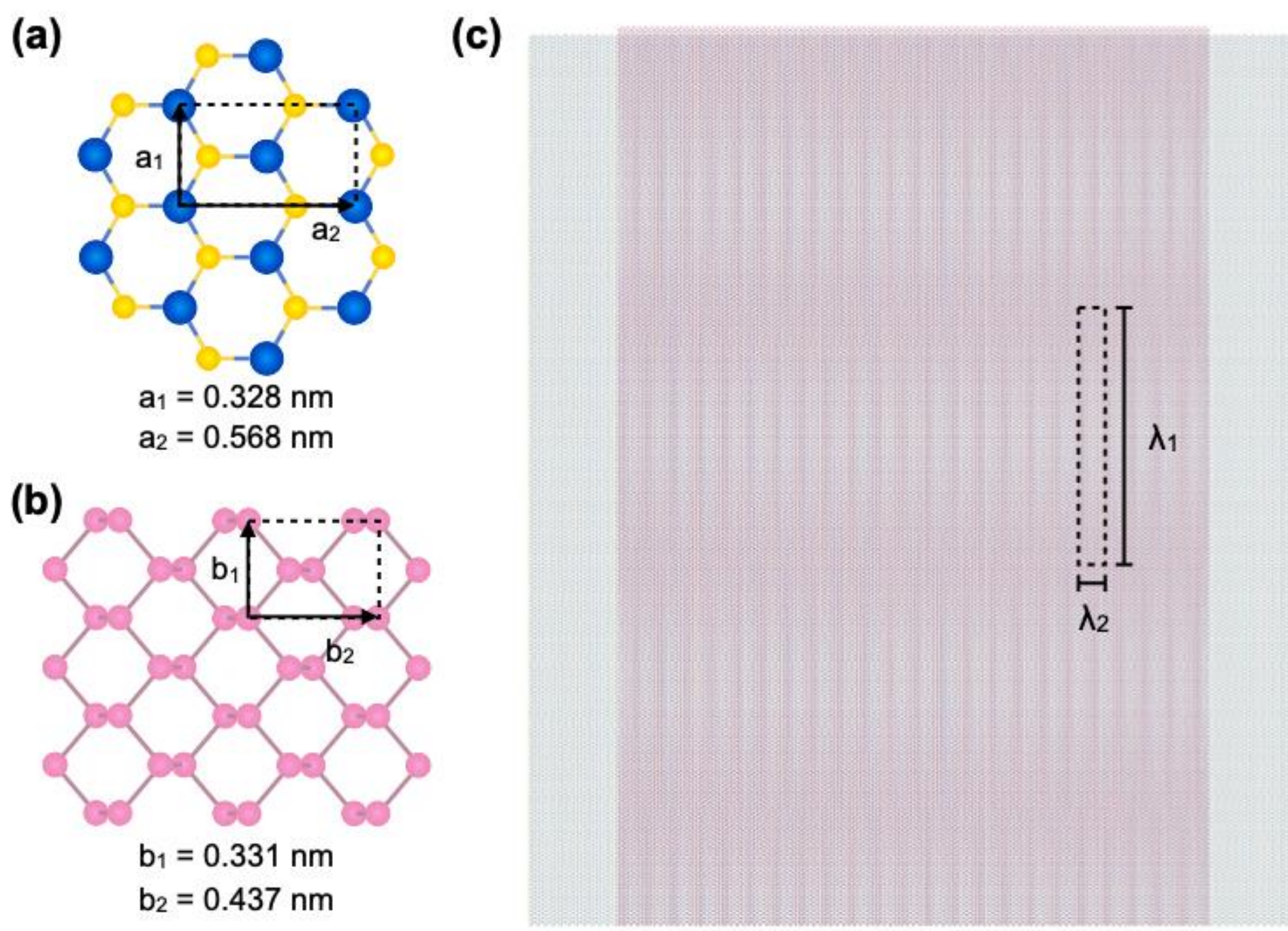

Figure S4. Stripe moiré superlattice in WSe_2/BP interface. (a), (b), Unit cells (indicated by the dashed rectangular) of WSe_2 (a) and BP (b) defined for moiré period estimation. The vectors are indicated by the black arrows. (c), Top view of WSe_2/ BP interface. Stripe moiré superlattice appears in 0° WSe_2/BP heterointerface. Two moiré periods, λ_1 and λ_2, are indicated by the black solid lines.

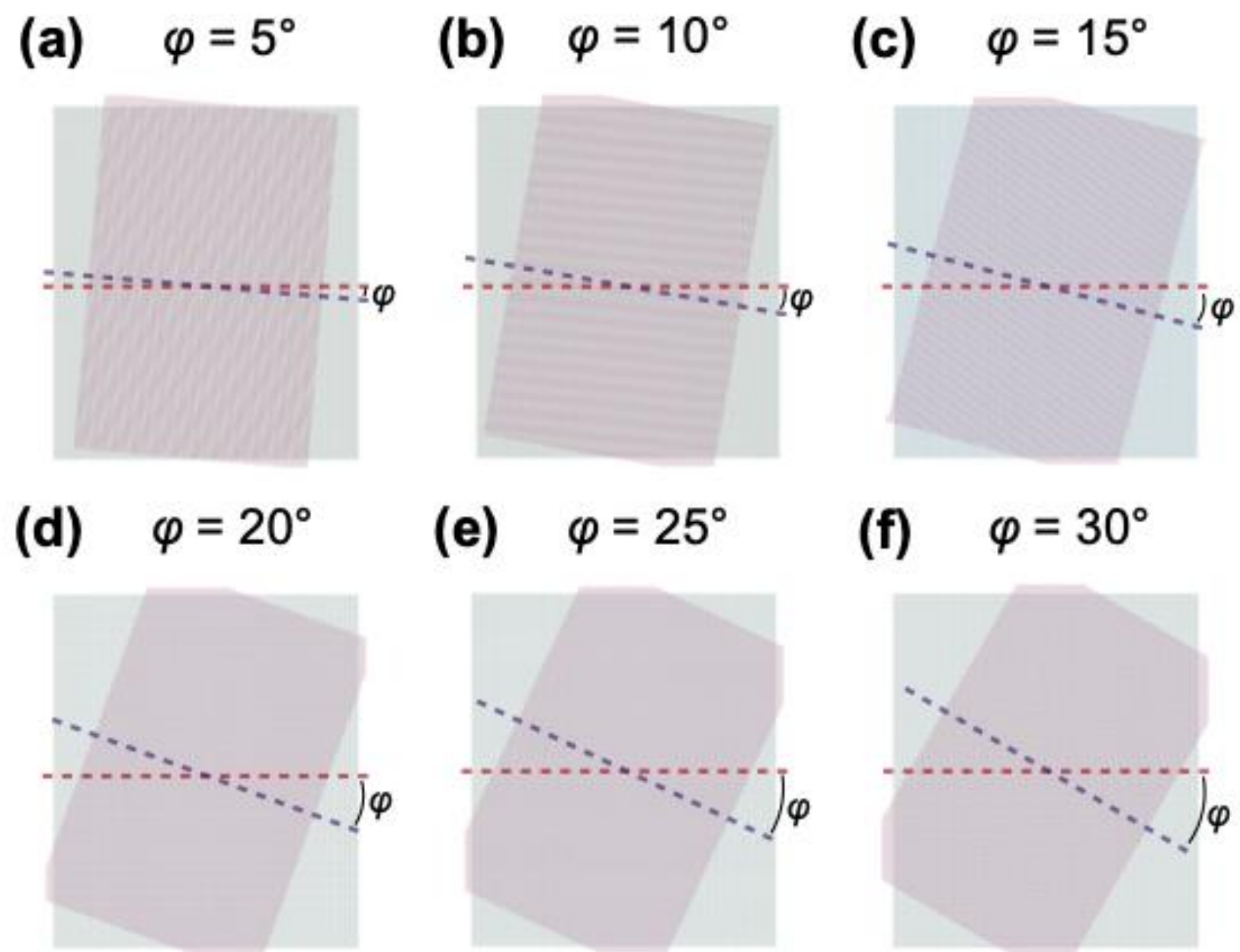

Figure S5. Stripe-like moiré superlattice in twisted WSe_2/BP interface. (a-f), Schematic illustrations of the stripe-like moiré superlattices in WSe_2/BP heterointerface with twist angles ranging from 5° to 30°.

5. The optical path of SHG measurement set-up

The schematics of the optical path of our homemade SHG set-up is shown in Figure S6. The measurements are performed under a normal-incidence geometry, which predominantly probes the in-plane nonlinear optical response. All measurements were performed in the ambient environment.

We consider the possible SHG generated by the half-wave plate placed before the 400 nm filter to be negligible in our measurements, for the following reasons. First, most of the reflected 800 nm fundamental light is removed by the harmonic separator, so the residual 800 nm component reaching the half-wave plate is expected to be very weak. It is therefore unlikely to generate a parasitic SHG signal of sufficient magnitude to affect the measured sample response. Second, if the half-wave plate were to generate an appreciable SHG signal, that contribution would be expected to appear over the entire illuminated area, rather than selectively from the sample region. When extracting the SHG polarization dependent patterns from the raw imaging data, we always subtract the background signal obtained from a nearby blank substrate region, which in principle generates no SHG signal. Therefore, even if a weak parasitic SHG contribution from the half-wave plate were present, it would be treated as part of the background and largely removed in the analysis.

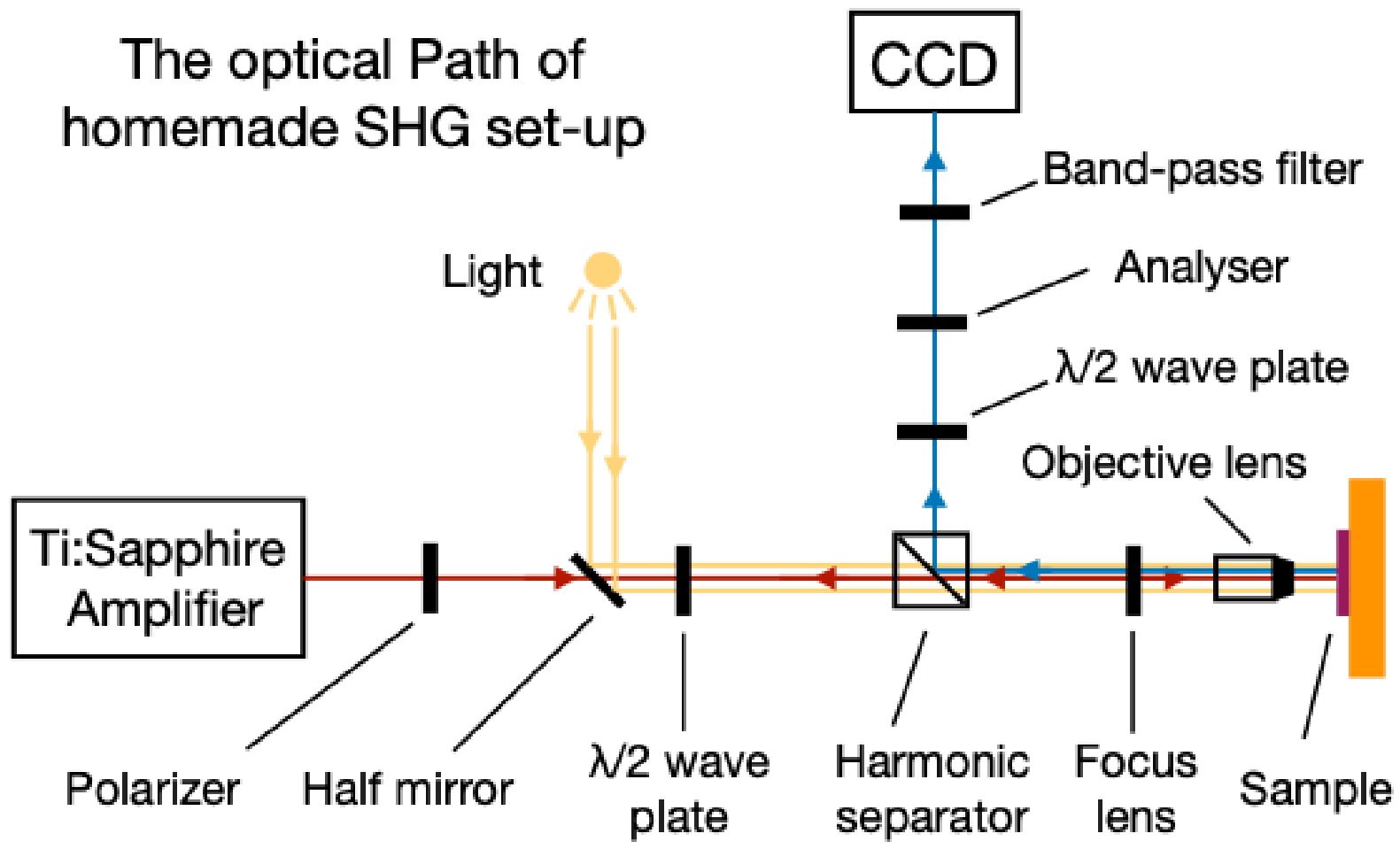

Figure S6. Optical path of homemade SHG measurement set-up. The excitation pulsed laser is 1.55 eV and the second harmonic signal is 3.1 eV.

6. Reproducibility of SHG response in WSe_2/BP interface

To confirm the reproducibility of SHG response from WSe_2/BP interface, we examined both the spatial dependence within a single WSe_2/BP sample (Figure S7) and the reproducibility across multiple samples with different BP thicknesses (Figure S8). For the spatial dependence, SHG signals were extracted from different regions of a single WSe_2/BP heterostructure, all of which exhibited consistent polarization-dependent patterns (Figure S7). To check the reproducibility across samples, we fabricated additional WSe_2/BP interfaces with 0° alignment (Figure S8 a-c). The BP flakes in these samples have different thicknesses. Nevertheless, all the observed SHG patterns (Figure S8 d-f) consistently showed peak enhancement along the expected polar direction, demonstrating robust reproducibility of the SHG response discussed in the main text.

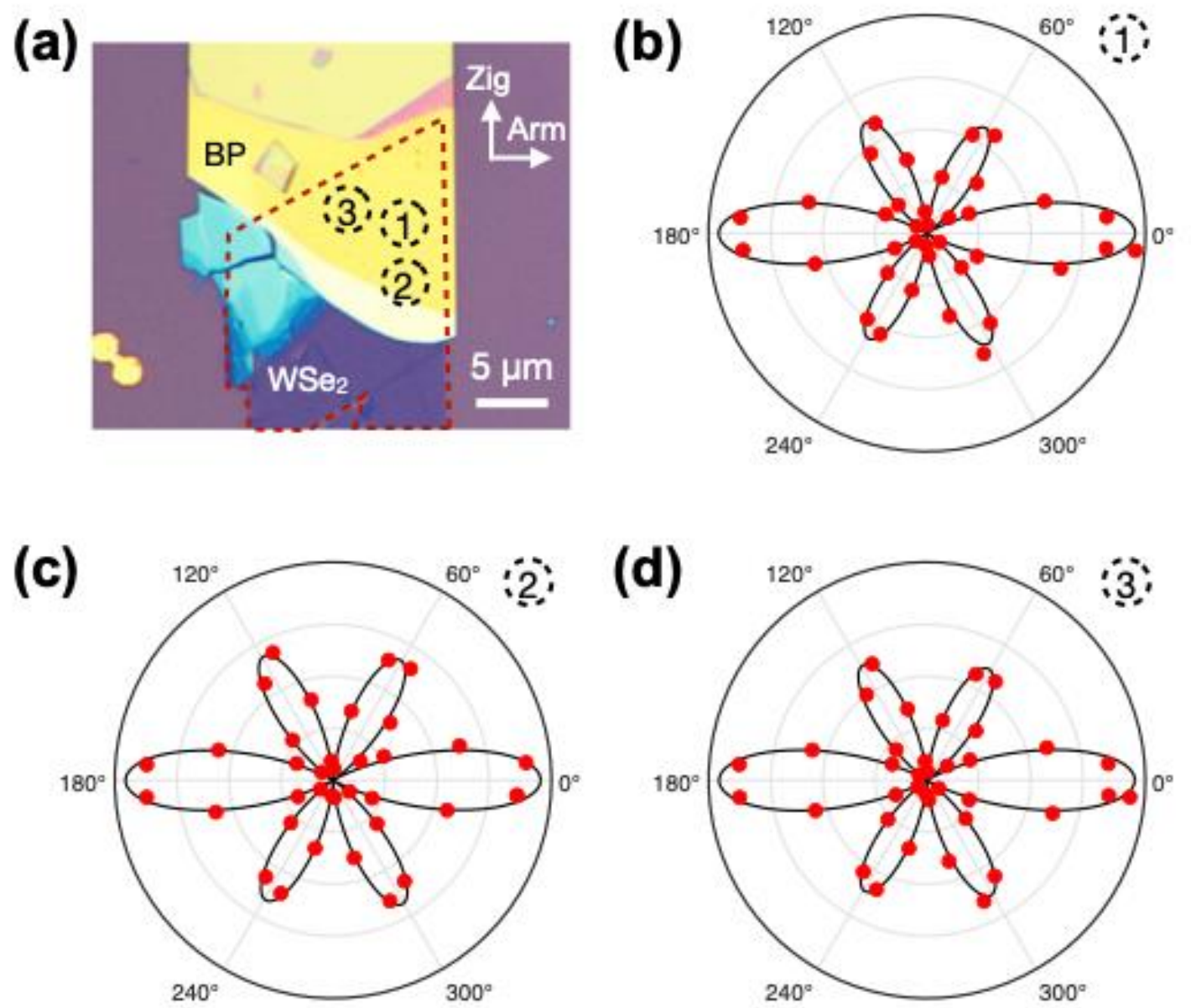

Figure S7. SHG reproducibility within a single WSe_2/BP sample. (a), The optical microscope image of the WSe_2/BP heterostructure. The red dashed line indicates the boundary of monolayer WSe_2 flake, and the black dashed circles represent the regions where the SHG signal were extracted. (b)-(d), Linear polarization-resolved SHG patterns obtained from the regions 1 to 3, respectively.

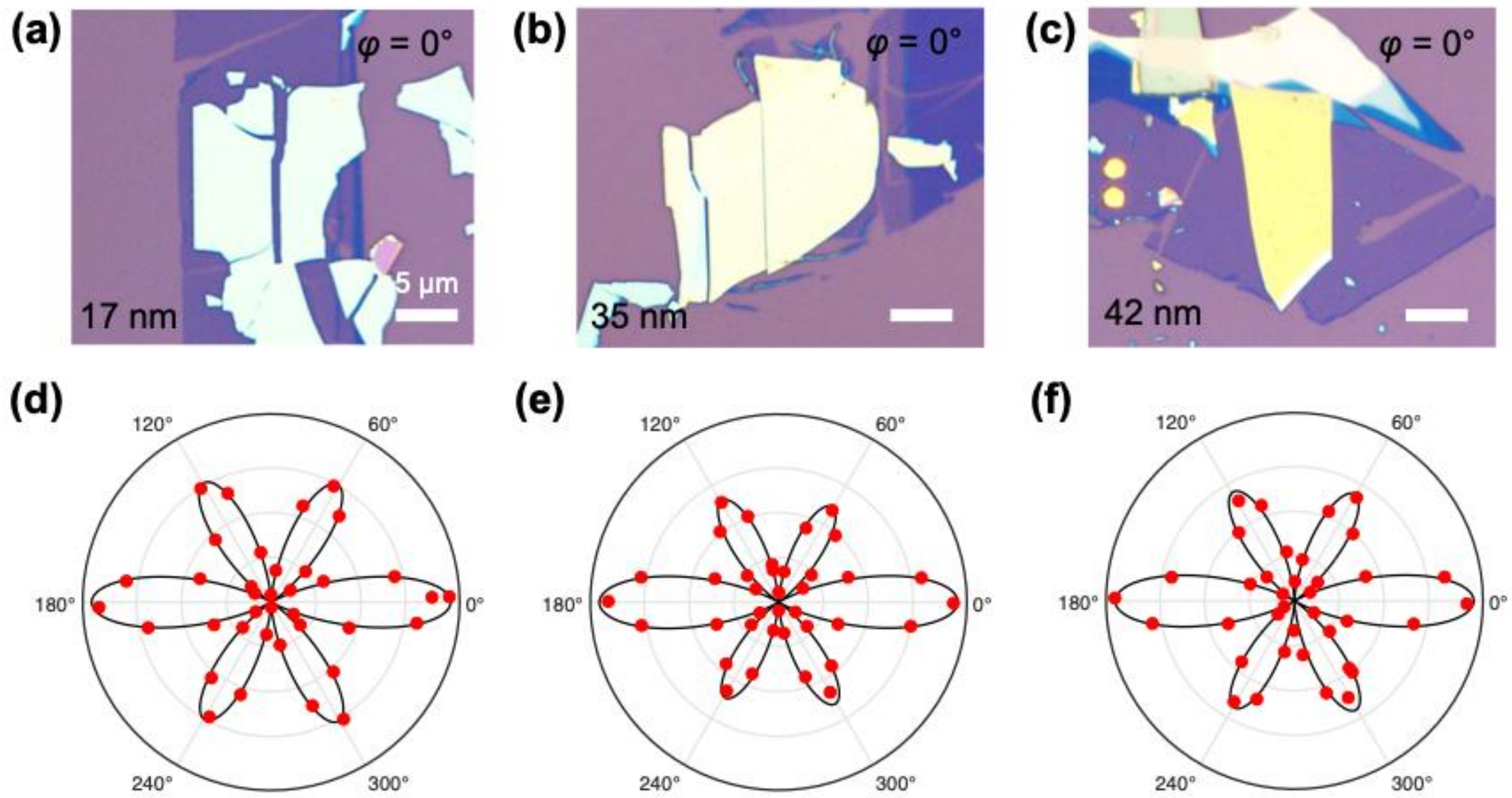

Figure S8. SHG reproducibility across multiple WSe_2/BP samples. (a)-(c), The optical microscope images of three WSe_2/BP heterointerface samples. The thickness of BP flake in each sample is 35 nm, 42 nm and 17 nm for (a), (b) and (c), respectively. (d)-(f), The polarization dependent SHG responses corresponding to WSe_2/BP samples in (a)-(c), respectively. All WSe_2/BP samples are well aligned (twist angle $\varphi = 0°$).

7. Interference effect: simulation method and additional simulation results

To compare the influence from the multiple interference effect and intrinsic electric polarization effect in SHG response of WSe_2/BP interface, we performed a simulation for SHG patterns by solving the nonlinear Maxwell equations[S4,5] within a WSe_2/IP/BP/SiO_2/Si multilayer structure, using realistic parameters listed in Table S1. Here, BP denotes black phosphorous, and IP refers to the WSe_2/BP interface, which hosts an emergent interface polarization. The IP layer was modeled as a 0.1-nm-thick virtual layer composed of 0.05 nm from WSe_2 (monolayer thickness: 0.65 nm) and 0.05 nm from BP (thickness: 40 nm), and its dielectric constant was approximated by averaging those of the two components as a first-order estimate.

The second-order nonlinear susceptibility ($\chi^{(2)}$) tensor of the WSe_2 monolayer follows Equation1 considering its point group symmetry $\bar{6}m2$. Here, $\chi^{(2)}_{xxx}$ is taken as the unit.

$$\text{WSe}_2\text{: } \chi^{(2)}_{xxx} = -\chi^{(2)}_{xyy} = -\chi^{(2)}_{yxy} = 1, \text{ others} = 0. \qquad (1)$$

For the IP layer with mirror symmetry along the armchair directions of aligned WSe_2 and BP (point group m), the general form of the $\chi^{(2)}$ tensor is given in Equation 2.

$$\text{IP: } \chi^{(2),\text{IP}}_{xxx} \neq 0,\ \chi^{(2),\text{IP}}_{xyy} \neq 0, \chi^{(2),\text{IP}}_{yxy} \neq 0, \text{ others} = 0. \qquad (2)$$

Since the emergent electric polarization at the interface scales with the interlayer coupling strength, the $\chi^{(2)}$ tensor of the IP layer is treated as a modulation of that of WSe_2 as following Equation 3.

$$\chi^{(2),\text{IP}}_{xxx} = 1 + \Delta\chi^{(2)}_{xxx},\ \chi^{(2),\text{IP}}_{xyy} = -1 + \Delta\chi^{(2)}_{xyy}\ ,\ \chi^{(2),\text{IP}}_{yxy} = -1 + \Delta\chi^{(2)}_{yxy} \qquad (3)$$

From the experimental observation, we find that $\Delta\chi_{yxy}^{(2)}$ is negligibly small, while $\Delta\chi_{xxx}^{(2)}$ and $\Delta\chi_{xyy}^{(2)}$ are finite values. To reproduce the SHG pattern observed in experiments (Figure 2c, main text), we consider the correction terms $\Delta\chi_{xxx}^{(2)} = 2$ and $\Delta\chi_{xyy}^{(2)} = \Delta\chi_{yxy}^{(2)} = 0$.

Figure S9 shows the BP thickness dependence of SHG pattern simulated without considering the interface polarization. In this case, the pattern geometry solely depends on the multiple interference (birefringence and interference) effect. The pattern shows suppressed 0° and 180° lobes at the BP thickness of 20 nm at first, and remains nearly unchanged as the BP thickness increase to 50 nm, followed by a notable enhancement of these lobes from 50 to 70 nm. Since the typical BP thickness used in the present work is 30 – 50 nm, corresponding to the patterns featured by suppressed 0° and 180° lobes, these simulation results indicate that birefringence and interference effect cannot account for the enhancement of those lobes in the patterns observed in our experiments (main text, Figure 1g). Furthermore, simulations of the twisted WSe_2/BP interfaces without considering interface polarization (Figure S10) also yield very different results from the experimental observation. At last, we carry out additional simulations by changing the BP dielectric constant at 400 nm (increase and decrease 20 % along zigzag and armchair directions) or changing BP thickness to 70 nm. The magnitude parameters as a function of the interface twist angle for these additional simulations are summarized in Figure S11. There was little change from the results shown in the main text, and a slight linear increase as a function of twist angle was observed, which cannot explain the trend observed in the experiment. These facts highlight the important contributions of interface electric polarization to the experimental SHG observations.

Layer	Thickness (nm)	Dielectric constant at 800 nm	Dielectric constant at 400 nm	Ref.
WSe_2	0.6	$14.28 + 3.04i$	$5.76 + 10.8i$	S6
BP	39.95	$\varepsilon_{\mathrm{x}}^{\mathrm{BP}} = 8.0 + 2.5i$<br>$\varepsilon_{\mathrm{y}}^{\mathrm{BP}} = 9.0 + 0.1i$	$\varepsilon_{\mathrm{x}}^{\mathrm{BP}} = 6.5 + 3.0i$<br>$\varepsilon_{\mathrm{y}}^{\mathrm{BP}} = 7.0 + 3.5i$	S7
SiO_2	300	$2.1339349 + 0.00381736i$	$2.177388 + 0.00796913i$	S8
Si	Semi-infinite	$13.46153327 + 0.03863824i$	$31.5116769 + 3.66923242i$	S9

Table S1. Parameters used for modelling. For BP, the dielectric constant at 520 nm has been used to approximate the values at 400 nm, due to the unavailability of data below 520 nm. x- and y-axes correspond to the armchair and zigzag directions of BP, respectively.

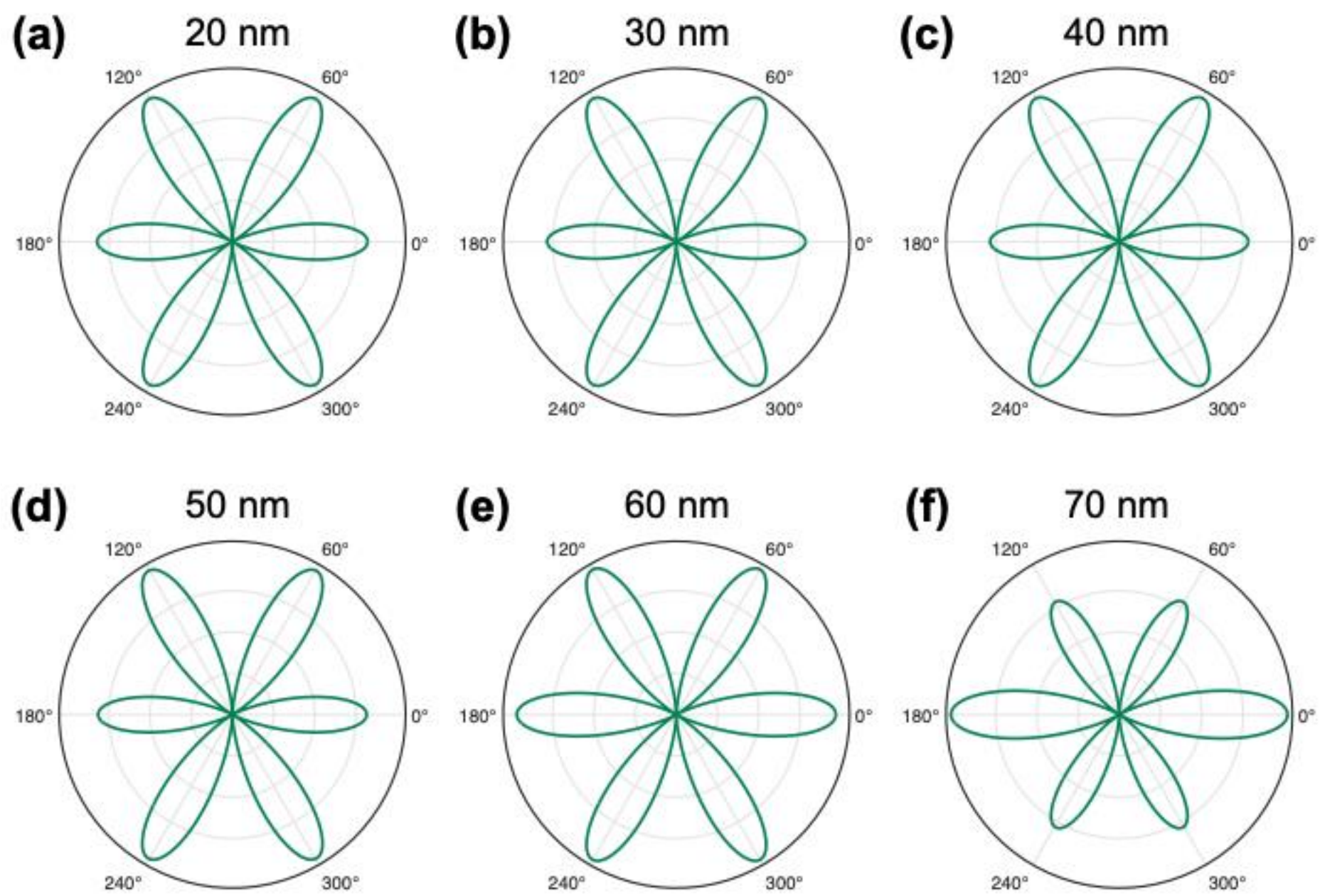

Figure S9. BP thickness dependence of SHG simulation for WSe_2/BP interface when only interference effects are considered. The simulated SHG polarization-resolved patterns of 0° WSe_2/BP interface as a function of BP flake thickness. Simulated patterns cannot explain the observed SHG patterns.

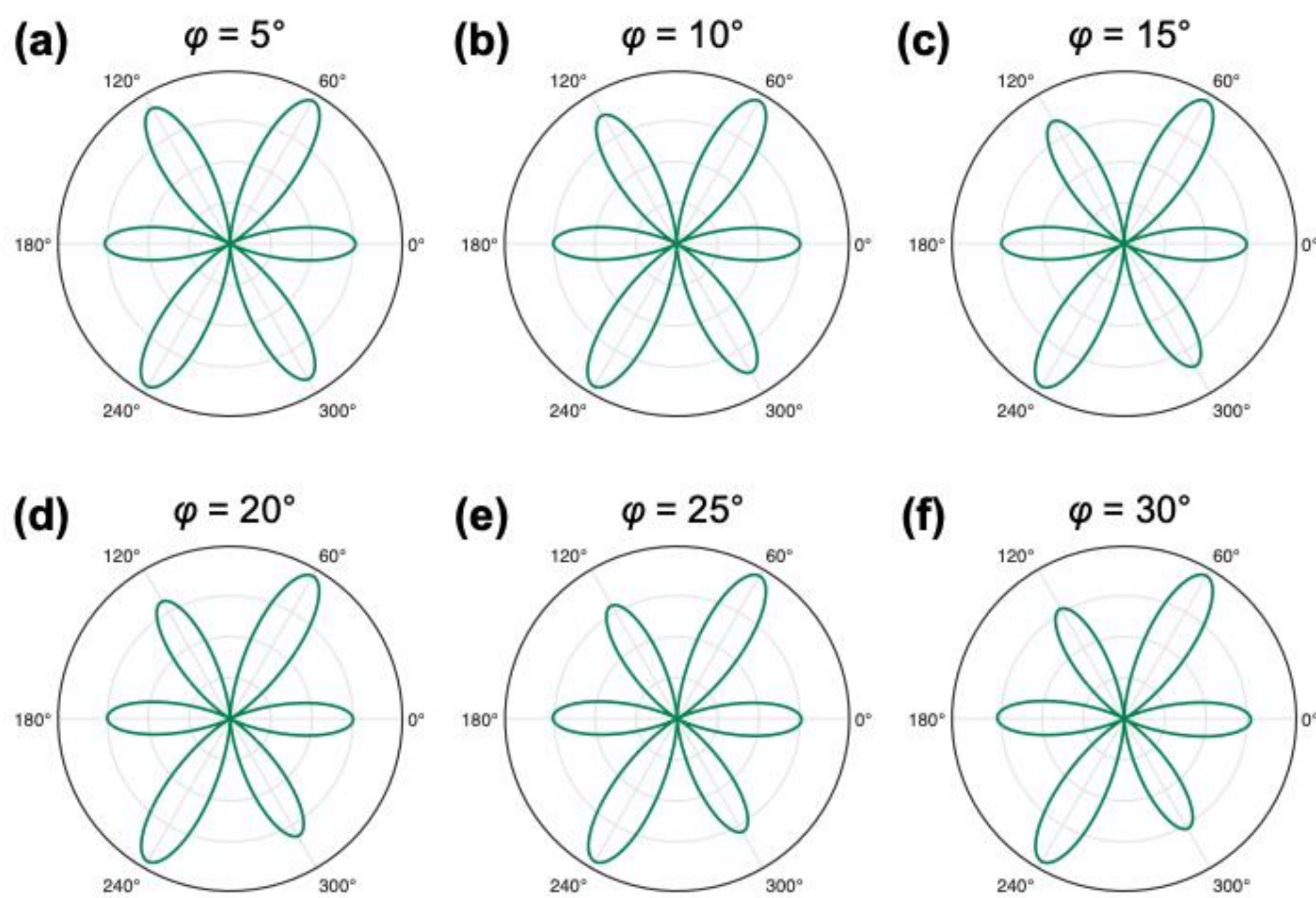

Figure S10. Interface twist angle dependence of SHG simulation for WSe_2/BP interface when only interference effects are considered. The simulated SHG polarization-resolved patterns of twisted WSe_2/BP interface as a function of the interface twist angle. The simulated pattern still cannot explain the observed SHG pattern.

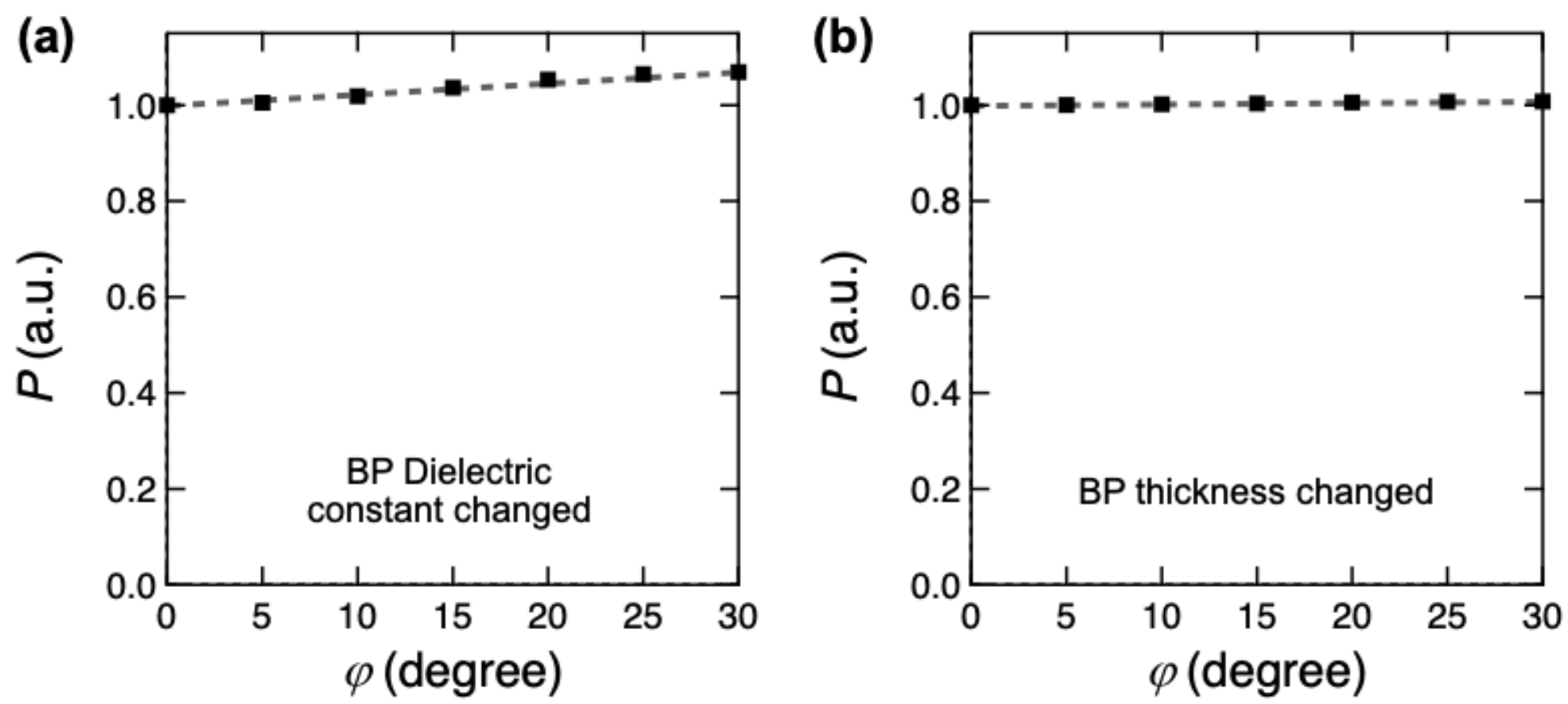

Figure S11. Interface twist angle dependence of magnitude parameter from the fitting of simulated SHG patterns when only interference effects are considered. Further SHG simulations (interference effect only) are carried out by changing the BP dielectric constant or BP thickness. The magnitude parameters as a function of interface twist angle are summarized in (a) and (b). In the simulation of (a), BP dielectric constants are changed to $\varepsilon_x = 0.8 \cdot \varepsilon_x^{\mathrm{BP}}$ (400 nm) for armchair direction and $\varepsilon_y = 1.2 \cdot \varepsilon_y^{\mathrm{BP}}$ (400 nm) for zigzag direction while BP thickness remains 40 nm. In the simulation of (b), BP thickness is changed to 70 nm while BP dielectric constants are unchanged.

8. Optical images of twisted WSe_2/BP samples

Pictures of twisted WSe_2/BP sample, whose SHG signals are discussed in the main text, are shown in Figure S12. From the sample pictures, stacking angle can be clearly estimated.

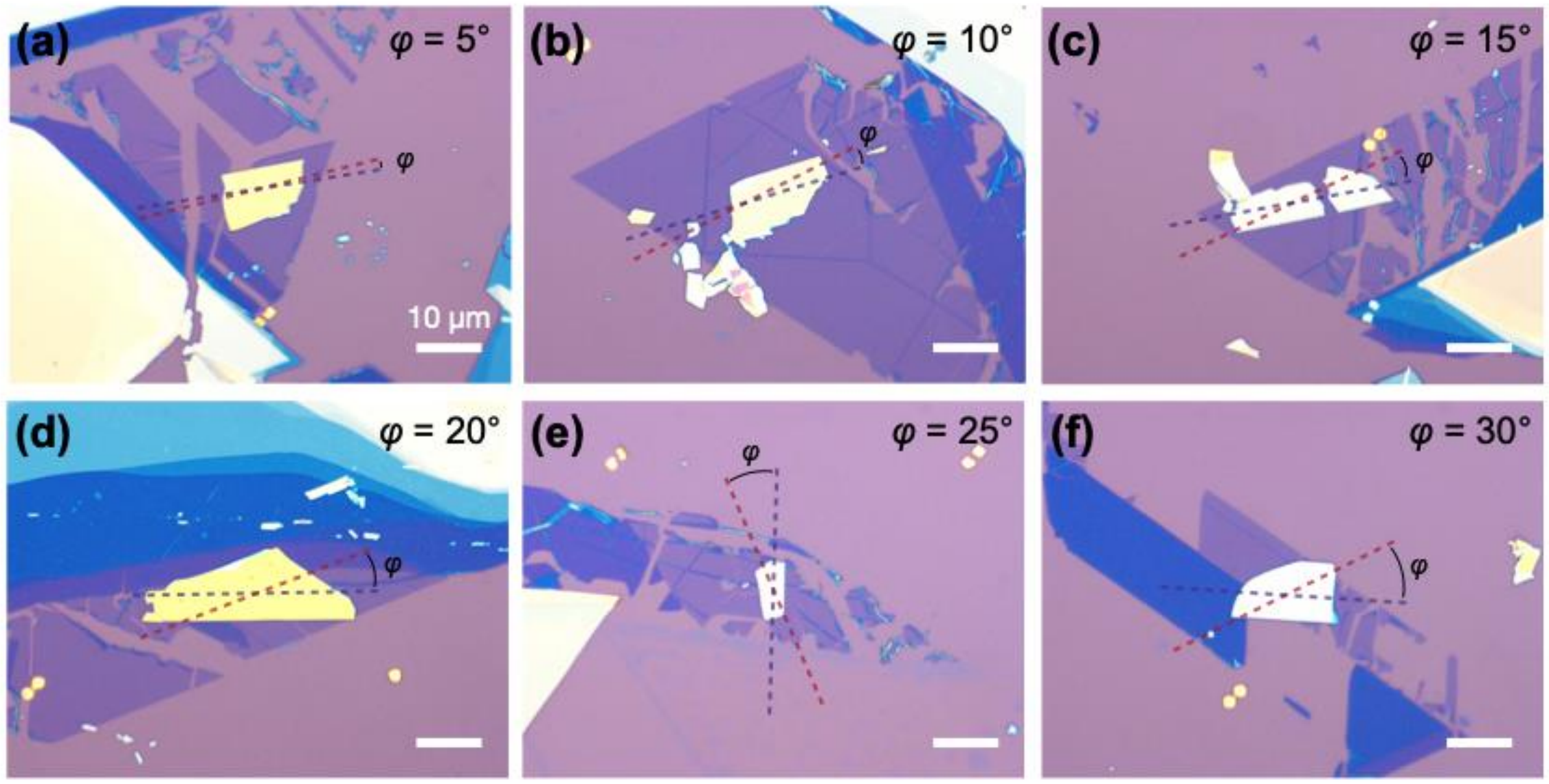

Figure S12. Optical images of twisted WSe_2/BP samples. (a)-(f), The optical microscope images of twisted WSe_2/BP heterointerface samples. The red and purple dashed lines indicate the armchair direction of monolayer WSe_2 and BP flakes, respectively. φ is the interface twist angle.

9. Transmission electron microscopy characterization on twisted WSe_2/BP interfaces

Further transmission electron microscopy (TEM) measurements are performed on twisted WSe_2/BP interface samples. In the twisted interface with a small twist angle of 2-3°, the stripe moiré pattern with a small period of ~5 nm is observed (see Figure S13a), which is consistent with the results from our moiré period estimation (3°). The small twist angle is also confirmed in the TEM diffraction pattern in Figure S13b (red and purple dashed lines), where the six-fold diffraction spots (highlighted by red circles) indicate WSe_2 lattice and the four-fold diffraction spots (highlighted by purple circles) correspond to BP lattice.

The real space TEM images of 10°, 20°, 30° twisted heterointerfaces are shown in Figure S14. However, no clear moiré superlattice is observed. The possible reason is that moiré periods of large twist angle WSe_2/BP interface become too small to notice. It is noted that the actual twist angles of 0°, 10°, 20°, 30° twisted interfaces are confirmed by the TEM diffraction patterns, as shown in Figure S15 (red and purple dashed lines indicating the crystal directions of WSe_2 and BP). The twist angle error is about 1° for all twisted WSe_2/BP interface samples.

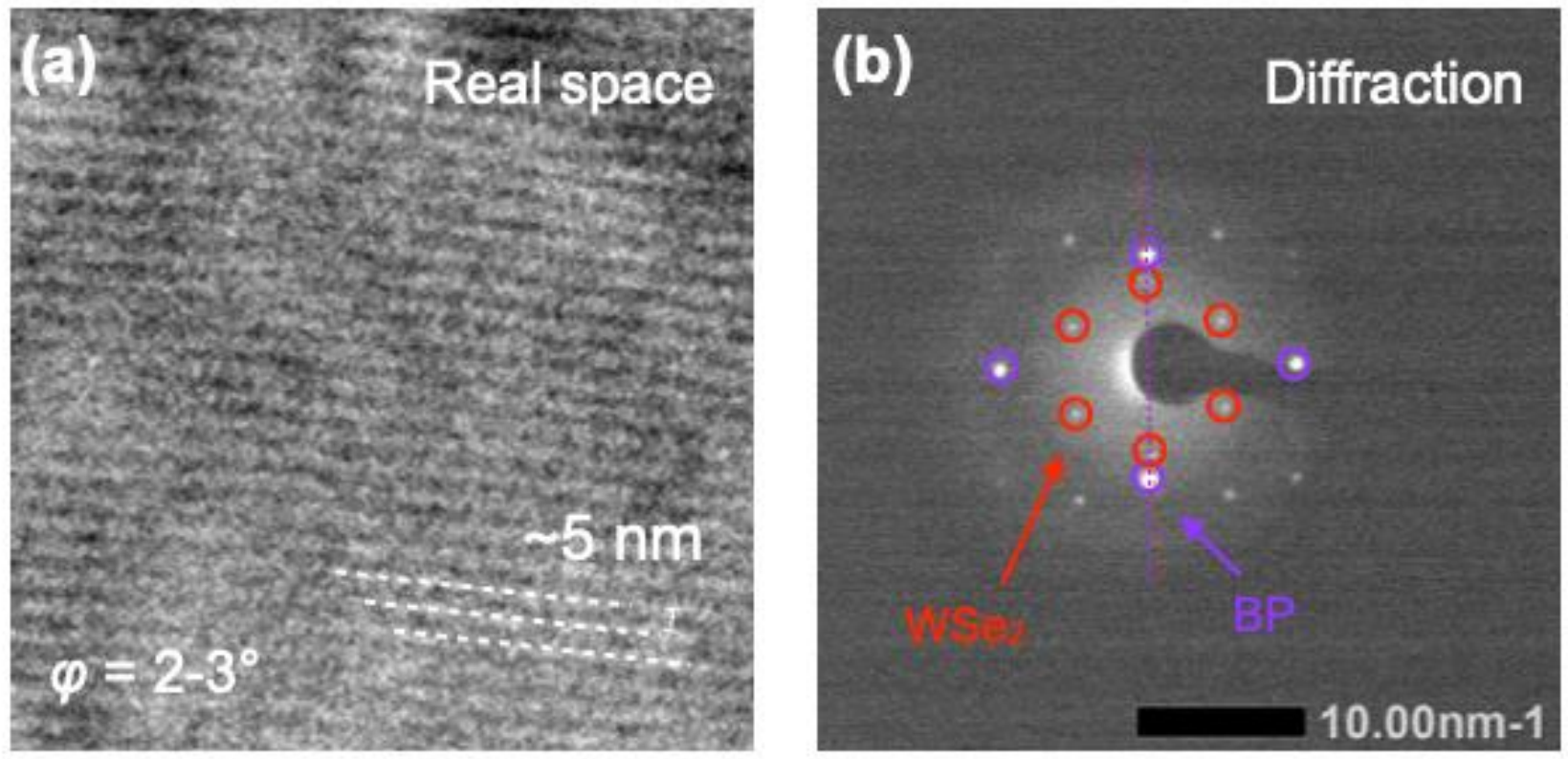

Figure S13. TEM real space and diffraction pattern of twisted WSe_2/BP interface with a small twist angle. (a), Real space TEM image of 2-3° twisted WSe_2/BP interfaces. The period of stripe moiré pattern indicated by the white dashed lines is ~ 5 nm. (b), TEM diffraction pattern of the same sample. The diffraction spots of WSe_2 and BP are highlighted by red and purple circles. The red and purple dashed lines indicate the WSe_2 and BP crystal axis.

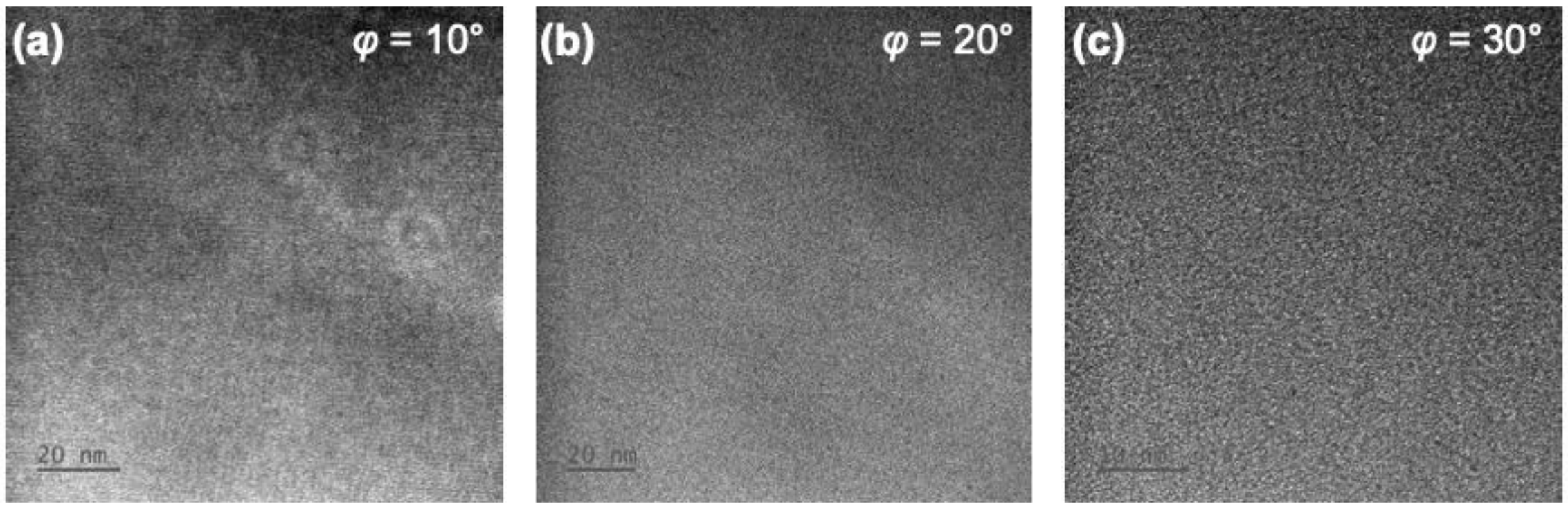

Figure S14. TEM real space images of twisted WSe_2/BP interfaces. (a)-(c), Real space TEM images of (a) 10°, (b) 20°, and (c) 30° twisted WSe_2/BP interfaces. No clear moiré superlattice is observed.

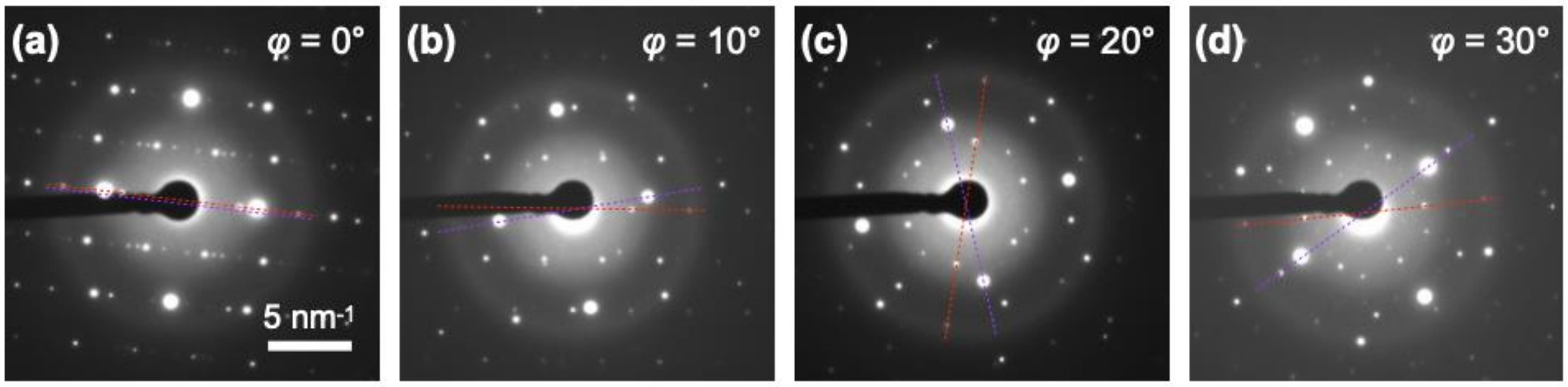

Figure S15. TEM diffraction patterns of twisted WSe_2/BP interfaces. (a)-(d), Electron diffraction patterns for selected area of (a) 0°, (b) 10°, (c) 20°, and (d) 30° twisted WSe_2/BP interfaces. The red and purple dashed lines indicate the crystal axis of WSe_2 and BP.

10. SHG response of WSe_2/BP interface in parallel and cross configurations

We performed SHG polarization dependence measurements on 0°, 5°, 15°, 20° and 30° twisted WSe_2/BP interfaces under parallel and cross configurations, where the linear polarization directions of the excitation laser and the detected SHG signal are either parallel or perpendicular to each other. The SHG results of 0° interface under parallel and cross configurations are shown in Figure S16a and S16f, respectively. In both cases, the SHG intensities exhibit almost perfectly mirror symmetric patterns along 0° direction, consistent with the expected polar axis. For the 30° twisted WSe_2/BP interface, the SHG patterns in both parallel (Figure S16e) and cross (Figure S16j) configurations are clearly mirror symmetric along the 60° direction, again aligned with the expected polar orientation. As to other twist angle interfaces, there is no obvious high symmetry direction in either parallel or cross configurations. These results confirm the robustness of the in-plane symmetry breaking, including electric polarization effect, observed from SHG across the different measurement configurations.

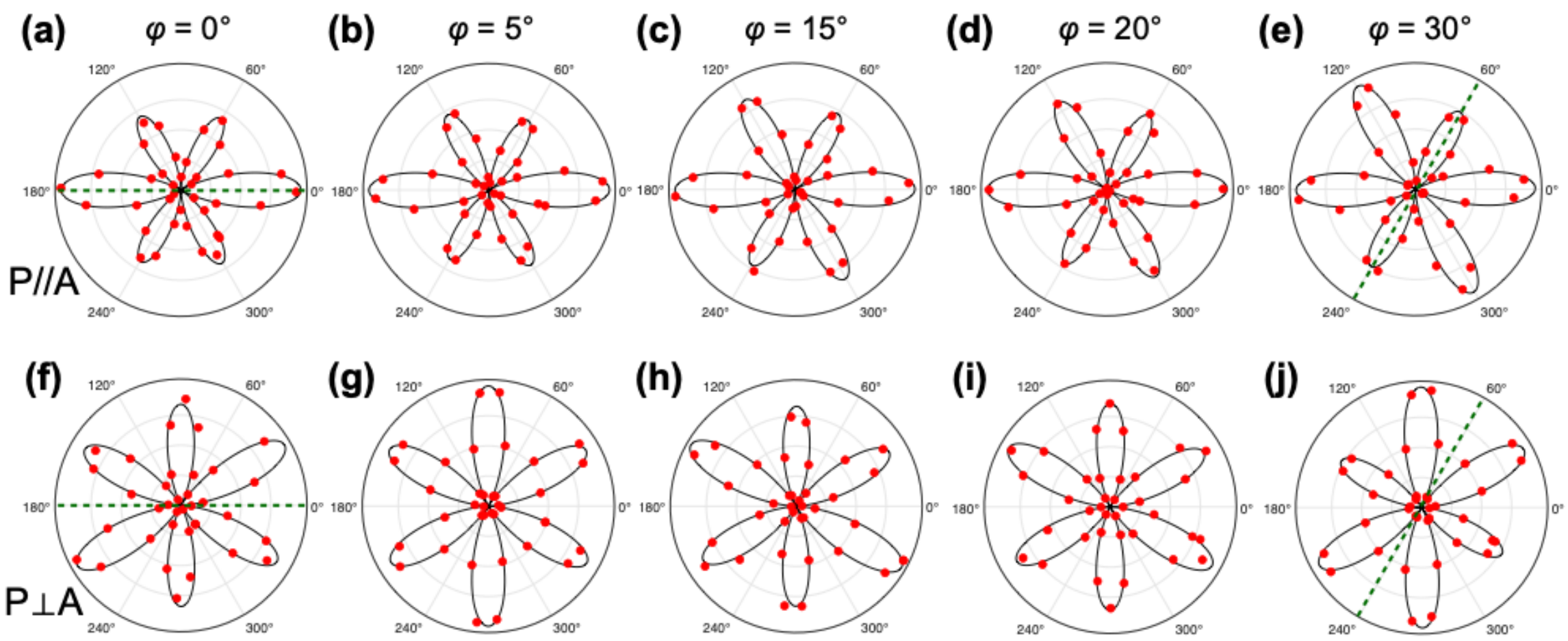

Figure S16. SHG response from WSe_2/BP interfaces under parallel and cross configurations. (a)-(e), The linear polarization dependent SHG patterns from 0°, 5°, 15°, 20° and 30° twisted WSe_2/BP interfaces measured in the parallel configuration. (f)-(j), The linear polarization dependent SHG patterns from 0°, 5°, 15°, 20° and 30° twisted WSe_2/BP interfaces measured in the cross configuration. The green dashed lines indicate the mirror symmetric direction of the SHG patterns, which are consistent with the expected polar directions.

11. Details about tight-binding model for twisted WSe_2/BP interface and calculation method

We consider the following effective tight-binding model for twisted WSe_2/BP interface:

$$H(\boldsymbol{k}) = \begin{pmatrix} m & \alpha\gamma^*(\boldsymbol{k}) & V(\boldsymbol{k}) \\ \alpha\gamma(\boldsymbol{k}) & -m & V(\boldsymbol{k}) \\ V(\boldsymbol{k}) & V(\boldsymbol{k}) & c_2(\boldsymbol{k}) \end{pmatrix},$$

$$\gamma(\boldsymbol{k}) = e^{ik_x} + 2e^{-ik_x/2}\cos\left(\frac{\sqrt{3}}{2}k_y\right),$$

$$c_2(\boldsymbol{k}) = t_x\cos\big(k_x\cos\theta - k_y\sin\theta\big) + t_y\cos\big(k_x\sin\theta + k_y\cos\theta\big) + \varepsilon.$$

The first 2 by 2 block represents the Hamiltonian for WSe_2 and the c_2 represents the band dispersion for BP. $V(\boldsymbol{k}) = V_0 e^{-|\boldsymbol{k}|}$ is the interlayer coupling between the two layers. The exponential dependence on $|\boldsymbol{k}|$ originates from the decay of the interlayer hopping amplitude in real space. Here, we adopt the parameters $m = 0.8$ eV, $\alpha = 0.3$ eV, $t_x = 0.5$ eV, $t_y = -0.1$ eV, $\varepsilon = -0.8$ eV, and $V_0 = 1$ eV. The interlayer coupling $V(\boldsymbol{k})$ between the two layers hybridizes electronic states of the tungsten (W) d-orbitals and those of the phosphorus (P) p-orbitals, giving rise to anisotropic electronic states. Due to the competition of three-fold and two-fold rotation symmetries of the two layers, the electronic charge distribution no longer preserves in-plane inversion equivalence, resulting in a finite in-plane polarization.

The SHG conductivity $\sigma^{abc}(\Omega)$ is computed using the formula,

$$\sigma^{abc}(\Omega) = -\frac{i\pi}{2\Omega^2}\sum_{\boldsymbol{k}mn}[v_{mn}^a w_{nm}^{bc}\delta(\omega_{nm}-2\Omega) + (w_{mn}^{ab}v_{nm}^c + w_{mn}^{ac}v_{nm}^b)\delta(\omega_{nm}-\Omega)]f_{mn}$$

$$-\frac{i\pi}{2\Omega^2}\sum_{\boldsymbol{k}mnp}\frac{v_{mn}^a\left(v_{np}^b v_{pm}^c + v_{np}^c v_{pm}^b\right)}{\omega_{pm}+\omega_{pn}}$$

$$\times\left[\delta\left(\omega_{pm}-\Omega\right)f_{mp} + \delta\left(\omega_{np}-\Omega\right)f_{pn} - 2\delta(\omega_{nm}-2\Omega)f_{mn}\right],$$

where Ω is the frequency of light, v^a is the a component of the velocity operator, $w^{ab} = \partial_{k_a}\partial_{k_b}H$, $\omega_{mn} = E_m - E_n$ and $f_{mn} = f(E_m) - f(E_n)$. The calculated polarization-resolved SHG patterns are shown in Figure S17.

The electric polarization of the interface P_α along the a direction is obtained from the polarization current originating from the interlayer coupling V_0 as

$$P_\alpha = \frac{e}{(2\pi)^2}\int_0^{V_0} dV' \int_{\mathrm{BZ}} d^2\boldsymbol{k}\, F_{\alpha V'}(\boldsymbol{k}),$$

$$F_{\alpha V_0}(\boldsymbol{k}) = i\sum_n\left[\frac{\partial}{\partial k_\alpha}\langle\psi_n(\boldsymbol{k})|\frac{\partial}{\partial V_0}|\psi_n(\boldsymbol{k})\rangle - \frac{\partial}{\partial V_0}\langle\psi_n(\boldsymbol{k})|\frac{\partial}{\partial k_\alpha}|\psi_n(\boldsymbol{k})\rangle\right],$$

where $|\psi_n(\boldsymbol{k})\rangle$ is the Bloch state of nth band. In the leading order, the hybridization effect between the two orbitals can be captured by the second order perturbation in terms of the interlayer coupling V. Thus, the induced in-plane polarization grows with the interlayer coupling as $\propto V^2$ as shown in the theoretical part of our previous study on the well-aligned WSe_2/BP heterointerface[S10].

We note that we perform the integration over the momentum k for the hexagonal area with vertices $\pm k_1, \pm k_2, \pm(k_1 - k_2)$ with $k_1 = (0, 4\pi/\sqrt{3})$, $k_2 = (2\pi, 2\pi/\sqrt{3})$. For precise computation of a physical quantity, one needs to average the physical quantity over the k area which contains multiple copies of Brillouin zones of the two layers and take into account folded

bands as many as possible[S11]. Here, we instead consider the unfolded band and perform the integration over the hexagon twice larger than the first Brillouin zone since the lattice constants of the two layers are close with each other and the interlayer coupling $V(\boldsymbol{k})$ decays quite quickly.

An order-of-magnitude estimate of the interfacial polarization can be obtained from the theoretical calculation. For instance, the previous work on the well-aligned WSe_2/BP heterointerface[S10]. Based on a tight-binding model calculation, the interfacial polarization along the polar direction was estimated to be approximately $\sim 7 \times 10^{-5} \mathrm{e}/\mathrm{Å}$, when an interlayer coupling parameter V_0 = 0.5 eV is assumed. This corresponds to approximately $0.112\ \mathrm{pC/m}$. Although this estimated polarization magnitude is considerably smaller than that of conventional bulk ferroelectrics, it is physically reasonable for an emergent interfacial polarization in a symmetry-mismatched van der Waals heterostructure.

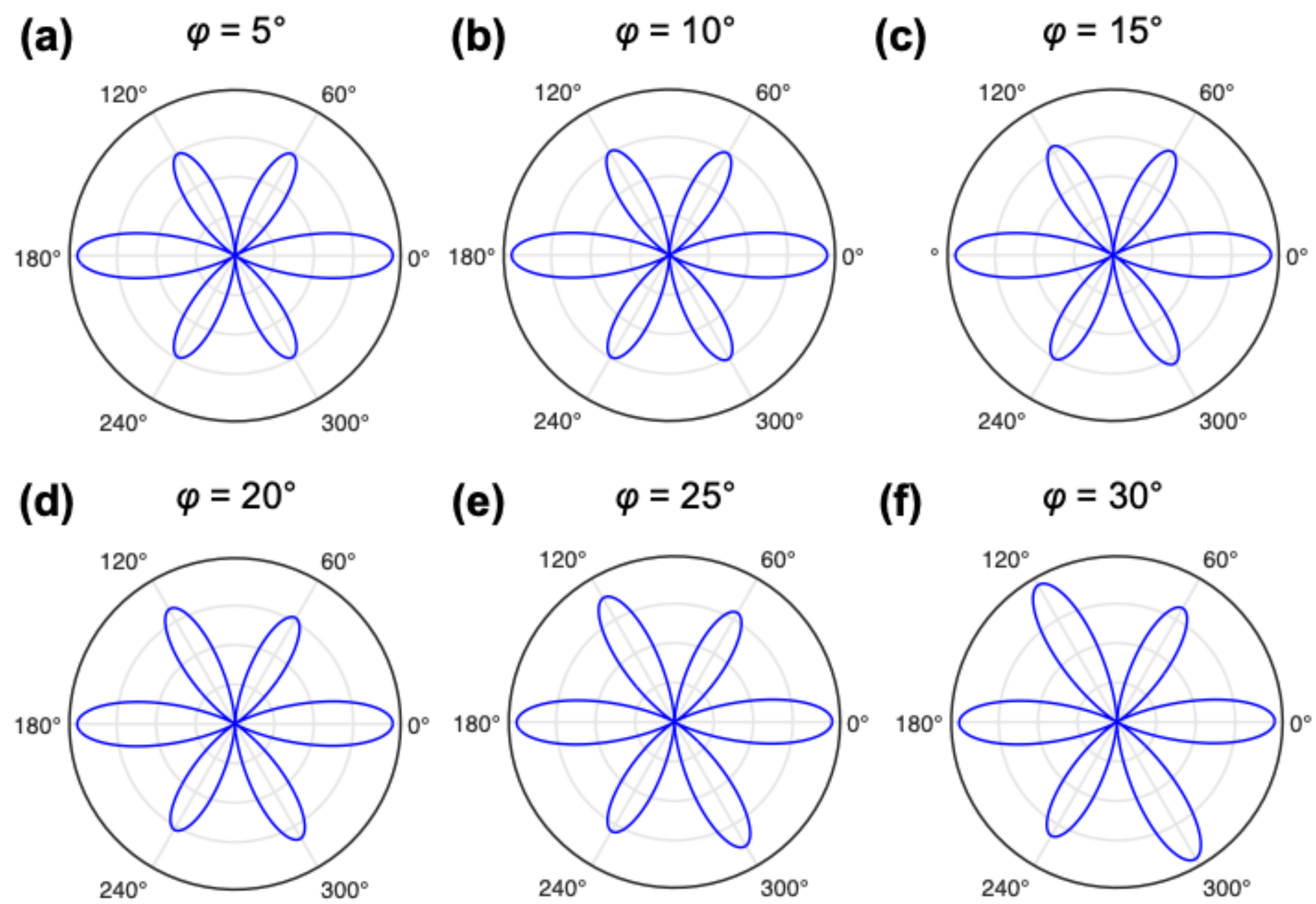

Figure S17. Calculated polarization-resolved SHG patterns based on the tight-binding model. (a)-(f) The calculated SHG polarization-resolved patterns of twisted WSe_2/BP interface as a function of the interface twist angle.

12. Photoluminescence response of WSe_2/BP interface

We performed low-temperature photoluminescence (PL) measurements on a 0° WSe_2/BP heterointerface at 5 K. The optical microscope image of the measured sample is shown in Figure S18a, where the red dashed line marks the monolayer WSe_2 boundary. The PL spectra measured from the interface region and the 1L WSe_2 region are summarized in Figure S18b. Compared with the 1L WSe_2 region, the PL intensity from the heterointerface is significantly suppressed, consistent with the previous report attributing this behavior to the interfacial charge transfer between WSe_2 and BP[S12]. In addition, the excitonic peaks near 1.72 eV and 1.74 eV observed in monolayer WSe_2 become strongly suppressed at the interface, while the dominant peak near 1.66 eV remains visible.

Interestingly, the interfacial PL spectrum exhibits additional fine structures, with several small sub-peaks appearing on the dominant peak (see the enlarged spectrum in Figure S18c). Such spectral features are absent in the smooth PL spectrum of 1L WSe_2 and may be related to the stripe moiré superlattice and the resulting spatial modulation of the interfacial potential. In twisted moiré systems, the periodically varying local atomic registry and interlayer coupling can generate a moiré potential landscape that modifies excitonic properties through local band renormalization, dielectric screening modulation, and exciton localization. As a result, moiré structures may induce additional excitonic fine structures, peak splitting, linewidth modulation, or spatially localized exciton states in PL spectra.

In the present symmetry-mismatched WSe_2/BP heterointerface, the stripe moiré geometry is highly anisotropic compared with conventional hexagonal moiré superlattices in twisted TMDC systems. Such anisotropic moiré modulation may further introduce direction-dependent excitonic

confinement, anisotropic oscillator strength, or anisotropic exciton transport. Meanwhile, the originally isotropic PL response from 1L TMDCs may also become anisotropic due to symmetry breaking at the heterointerface, according to the previous study on symmetry-mismatched MoS_2/SiP interfaces[S13]. Therefore, the observed fine structures in the interface PL spectrum may reflect the influence of the stripe-like moiré potential on the excitonic landscape. However, a detailed understanding of these features would require further systematic studies, such as polarization-resolved PL, differential reflectance spectroscopy, or spatially resolved optical measurements.

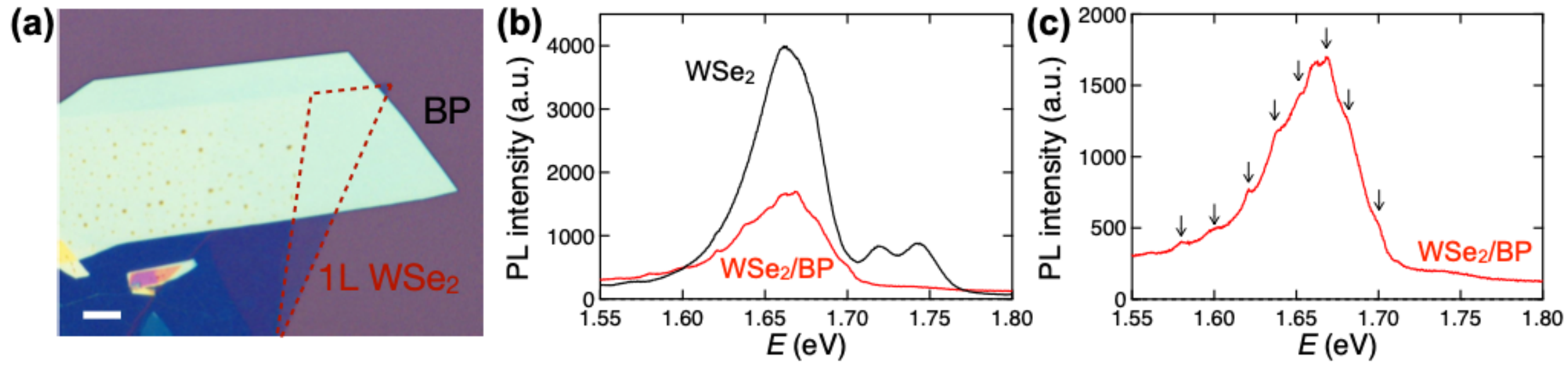

Figure S18. (a) Optical microscope image of the 0° WSe_2/BP device. The red dashed line marks the boundary of the 1L WSe_2 region. (b) The PL spectra measured from the WSe_2/BP heterointerface and 1L WSe_2 region. (c) Enlarged view of the PL spectrum from WSe_2/BP heterointerface, highlighting the fine spectral features on the dominant peak. The black arrows indicate the positions of the sub-peaks. All measurements were performed at 5 K.

13. Raman characterization of the crystal orientation in black phosphorus

The crystal orientation of black phosphorus was first determined by the optical images, where long and straight edges are assumed to be zigzag direction. This preliminary determination was further confirmed by angle-resolved Raman spectroscopy. The Raman spectrum of BP is shown in Figure S19a from a representative sample (Figure S19b). Among the three characteristic peaks of BP, the intensity ratio between A^1_g and A^2_g modes varies as the polarization direction of the incident laser is rotated, due to the anisotropic vibration of atomic bonds in BP. As shown in Figure S19c, the ratio of peak intensity (A^2_g /A^1_g) reaches its maximum, when the linear polarization is aligned parallel to the armchair direction of the BP flake.

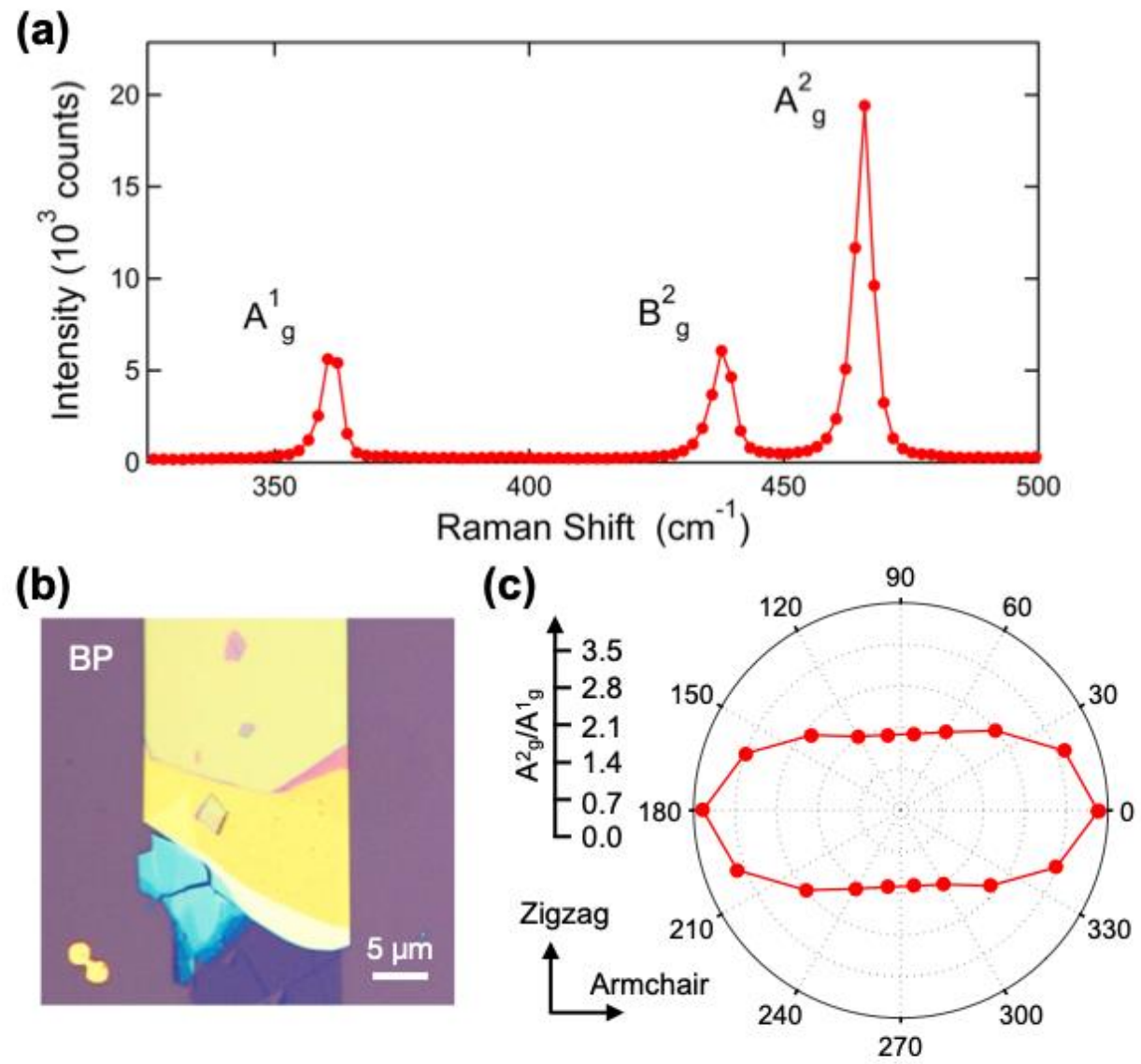

Figure S19. Angle-resolved Raman spectroscopy of BP flake. (a), Raman spectrum of BP flake. (b), The optical image of the measured BP flake. (c), The linear polarization dependence of the peak $A^2{}_g$ /$A^1{}_g$ intensity ratio. The black arrows indicate the BP crystal orientation for both the BP optical image (b) and the polar plot of the peak intensity ratio (c).

14. Discussion about strain in WSe_2/BP heterointerface

In the WSe_2/BP heterointerface studied here, two types of strain should be discussed: extrinsic strain unintentionally introduced during fabrication process, and intrinsic microscopic strain associated with the symmetry-mismatched interface itself.

The influence of extrinsic strain introduced during the transfer and fabrication process is expected to be limited in the present samples. To minimize residual fabrication-induced strain, the heterostructures were annealed at 220 °C for 2 hours in an N_2 atmosphere after sample assembly (see Experimental details section). More importantly, the experimentally observed SHG evolution exhibits a systematic dependence on twist angle across multiple samples, which is inconsistent with the random and sample-dependent nature typically expected for extrinsic strain effects. This fact further rules out the possible contribution of extrinsic strain.

In contrast, intrinsic microscopic strain naturally emerges from the lattice mismatch and possible structural reconstruction at the symmetry-mismatched WSe_2/BP interface. The strong in-plane anisotropy of BP can induce local symmetry lowering and anisotropic modification of the nonlinear optical susceptibility tensor. From this perspective, intrinsic strain should not be viewed as an independent competing mechanism, but rather as one microscopic manifestation of the broader interface-coupling and symmetry-engineering effects discussed in this work. The interplay between intrinsic strain, moiré superlattice, and emergent interfacial functionalities has also been also discussed in the previous study[S13].

Overall, the systematic twist angle dependence of SHG patterns, together with the consistency between experimental observations and theoretical modelling, suggests that the dominant physics

originates from twist-controlled interfacial symmetry breaking and polarization evolution, rather than random/sample-dependent extrinsic strain effects.

REFERENCES

[S1] Castellanos-Gomez, A.; Buscema, M.; Molenaar, R.; Singh, V.; Janssen, L.; van der Zant, H. S. J.; Steele, G. A. Deterministic transfer of two-dimensional materials by all-dry viscoelastic stamping. *2D Mater.* **2014**, 1, 011002

[S2] Guo, Y.; Liu, C.; Yin, Q.; Wei, C.; Lin, S.; Hoffman, T. B.; Zhao, Y.; Edgar, J. H.; Chen, Q.; Lau, S. P.; Dai, J.; Yao, H.; Wong, H.-S. P.; Chai, Y. Distinctive in-plane cleavage behaviors of two-dimensional layered materials. *ACS Nano* **2016**, 10, 8980–8988

[S3] Wu, J.; Mao, N.; Xie, L.; Xu, H.; Zhang, J.; Identifying the crystalline orientation of black phosphorus using angle-resolved polarized Raman spectroscopy. *Angew. Chem. Int. Ed.* **2015**, 54, 2366–2369 (2015).

[S4] Bloembergen, N.; Pershan, P. S. Light Waves at the Boundary of Nonlinear Media, *Phys. Rev.* **1962**, 128, 606

[S5] Berreman, D. W. *Optics in Stratified and Anisotropic Media: 4×4-Matrix Formulation*, *J. Opt. Soc. Am.* **1972**, 62, 502

[S6] Hsu, C.; Frisenda, R.; Schmidt, R.; Arora, A.; de Vasconcellos, S. M.; Bratschitsch, R.; J. van der Zant, H. S.; Castellanos-Gomez, A. Thickness-Dependent Refractive Index of 1L, 2L, and 3L MoS_2, $MoSe_2$, WS_2 and WSe_2, *Adv. Opt. Mater.* **2019**, 7, 1900239

[S7] Wang, X.; Lan, S. Optical propertie of black phosphorus. *Adv. Opt. Photonics* **2016**, 8 (4), 618-655

[S8] Rodríguez-de Marcos, L. V.; Larruquert, J. I.; Méndez, J. A.; Aznárez, J. A. Self-Consistent Optical Constants of SiO_2 and Ta_2O_5 Films, *Opt. Mater. Express* **2016**, 6, 3622

[S9] Schinke, C.; Christian Peest, P.; Schmidt, J.; Brendel, R.; Bothe, K.; Vogt, M. R.; Kröger, I.; Winter, S.; Schirmacher, A.; Lim, S.; Nguyen, H. T.; MacDonald, D. Uncertainty Analysis for the Coefficient of Band-to-Band Absorption of Crystalline Silicon, *AIP Adv*. **2015**, 5, 067168

[S10] Akamatsu, T.; Ideue, T.; Zhou, L.; Dong, Y.; Kitamura, S.; Yoshii, M.; Yang, D.; Onga, M.; Nakagawa, Y.; Watanabe, K.; Taniguchi, T.; Laurienzo, J.; Huang, J.; Ye, Z.; Morimoto, T.; Yuan, H.; Iwasa, Y. A van der Waals interface that creates in-plane polarization and a spontaneous photovoltaic effect. *Science* **2021**, 372 (6537), 68-72

[S11] Yoshii, M.; Kitamura, S.; Morimoto, T. Brillouin zone folding method for quasiperiodic superconductivity in multilayer systems: Application to electronic structure and optical responses. *Phys. Rev. B* **2025**, 111, 024206

[S12] Zong, X.; Hu, H.; Ouyang, G.; Wang, J.; Shi, R.; Zhang, L.; Zeng, Q.; Zhu, C.; Chen, S.; Cheng, C.; Wang, B.; Zhang, H.; Liu, Z.; Huang, W.; Wang, T.; Wang, L.; Chen, X. Black Phosphorus-based van der Waals Heterostructures for Mid-infrared Light-emission Applications, *Light Sci. Appl.* **2020**, 9, 114

[S13] Li, Z.; Huang, J.; Zhou, L.; Xu, Z.; Qin, F.; Chen, P.; Sun, X.; Liu, G.; Sui, C.; Qiu, C.; Lu, Y.; Gou, H.; Xi, X.; Ideue, T.; Tang, P.; Iwasa, Y.; Yuan, H. An Anisotropic van der Waals Dielectric for Symmetry Engineering in Functionalized Heterointerfaces, *Nat. Commun.* **2023**, 14, 5568
